# Severe acute malnutrition treatment delivered by low-literate community health workers in South Sudan: A prospective cohort study

**DOI:** 10.7189/jogh.10.010421

**Published:** 2020-06

**Authors:** Naoko Kozuki, Elburg Van Boetzelaer, Casie Tesfai, Annie Zhou

**Affiliations:** 1 International Rescue Committee, Washington, D.C., USA; 2 International Rescue Committee, South Sudan, Panthou, South Sudan; 3 International Rescue Committee, New York, New York, USA

## Abstract

**Background:**

Community health worker (CHW)-delivered acute malnutrition treatment programs have been tested previously, but not with low-literate/-numerate cadres who operate in areas with the highest malnutrition burden and under-five mortality rates. The International Rescue Committee developed low-literacy-adapted tools and treatment protocol to enable low-literate/-numerate community-based distributors (CBD, the CHW cadre in South Sudan) to treat children for severe acute malnutrition (SAM) in their communities.

**Methods:**

We conducted a prospective cohort study in March-September 2017, with 44 CBDs enrolling a total of 308 SAM children into treatment in their communities. Child treatment outcomes and length of treatment were documented. Uncomplicated SAM cases, defined for our study as children with mid-upper arm circumference (MUAC) of 90 to <115 mm or bilateral pitting oedema, without any medical complications, were treated for up to 16 weeks, and were considered fully recovered when they reached MUAC≥125 mm for two consecutive weeks.

**Results:**

The recovery rate from the severe to the moderate acute malnutrition (MAM) cut-off of MUAC 115 mm was 91% (95% confidence interval (CI) = 88%-95%). The median length of treatment was five weeks. The recovery rate of children from SAM to full recovery was 75% (95% CI = 69%-81%). The median time to full recovery was eight weeks. The recovery rates reported here exclude children referred for care from the denominator, per standard reporting of acute malnutrition treatment recovery rates. When the data were compared against routine monitoring and evaluation data from nearby static clinics, children treated by CBDs appeared to have improved continuity of care and shorter time to recovery.

**Conclusions:**

The recovery rate for SAM children enrolled in acute malnutrition treatment by low-literate CBDs shows promise that deploying CHWs to treat SAM in areas with high prevalence and low treatment access may lead to higher recovery, better continuity of care in the transition between SAM and MAM, and shorter treatment time. Proper adaptations of tools and protocols can empower CHW cadres with low literacy and numeracy to successfully complete treatment steps. Key questions of scalability and cost-effectiveness remain.

The World Health Organization (WHO) estimates that 17 million under-five children suffer from severe acute malnutrition (SAM) [1]. As with other health services, access to treatment for acute malnutrition is a major issue in low-resource contexts; reported barriers include distance, opportunity cost to caregivers, and lack of recognition of malnutrition, with insecurity as an additional concern in fragile contexts [2-4].

Community-based service delivery models have been used to increase access to health care, one example being the integrated community case management (iCCM) of childhood illness strategy that equips community health workers (CHW) to deliver treatment in their communities for the major causes of death in under-five children: pneumonia, diarrhea, and malaria [5]. Similar models have been tested to deliver acute malnutrition treatment for uncomplicated SAM cases. One study conducted in Malawi demonstrated that treatment outcomes did not differ between CHWs and medical professionals at outpatient clinics [6]. A pilot study in Bangladesh employed CHWs to treat uncomplicated acute malnutrition in their home communities, and approximately 90% of the 55 CHWs partaking in the study passed a quality of care assessment at a pre-determined acceptable score of 90% [7]. However, a recent review highlighted that previous studies all utilized literate CHW cadres [8]; thus the findings are not necessarily applicable to contexts where human resource capacity is very limited. It is unclear how appropriate this treatment delivery model is when utilizing CHWs who have limited literacy and numeracy capacity.

The International Rescue Committee (IRC) developed tools and a protocol for low-literate CHWs to treat uncomplicated SAM cases in their communities. The IRC piloted the tools and protocol in Northern Bahr El Ghazal State, South Sudan, deploying low-literate community-based distributors (CBD, the CHW cadre in South Sudan) to deliver treatment in their community. We conducted a prospective cohort study to track the treatment outcomes of children treated under this model. The performance of the low-literate CHWs in adhering to the treatment protocol is published elsewhere [9].

## METHODS

**Figure 1:**
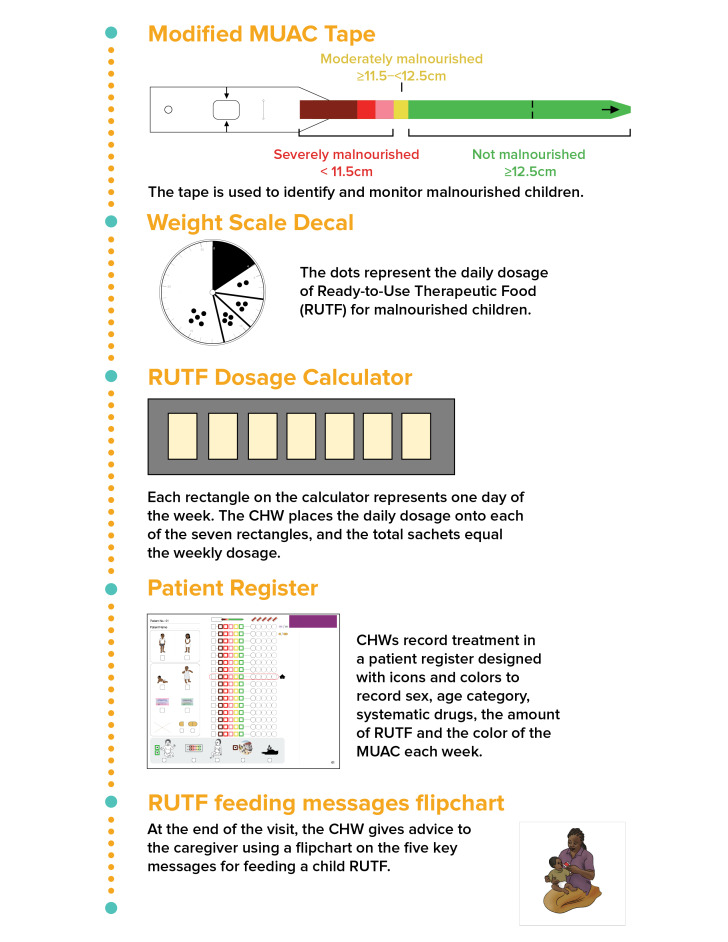
Low-literacy-adapted job aids and tools. Reproduced from [9], with permission.

The IRC began developing tools and a protocol that do not require literacy or numeracy for treating uncomplicated SAM cases in 2015, using a human-centered design approach. The first round of development occurred in Chad, Mali, and South Sudan, where local CHWs and IRC’s nutrition staff provided input. The additional rounds were used to refine and test generalizability in other contexts: round 2 (a design workshop in Mali, with additional attendees from Chad, Niger, and South Sudan), round 3 (India), round 4 (South Sudan), and round 5 (South Sudan). The last round was completed in November 2016. More detailed description of this process is available elsewhere [10]. The process resulted in five adapted tools: 1) patient register, 2) modified mid-upper arm circumference (MUAC) tape, 3) weight scale decal to identify daily dosage of treatment, 4) weekly dosage calculator, and 5) visual counselling cards (**Figure 1**) [9]. Detailed descriptions of each tool and the treatment protocol are available in Appendix S1 of the **Online Supplementary Document**.

The pilot was conducted in Aweil South County, Northern Bahr El Ghazal State, South Sudan, between March-September 2017. CBDs selected for study participation had worked for an IRC-supported integrated community case management (iCCM) of childhood illness program and were already trained to treat children under-five for uncomplicated pneumonia, diarrhea, and malaria. The CBDs received 100 USD per month as an incentive through the iCCM program, per national policy. Each CBD served 35 or more households. We note here that the iCCM program became inactive a month before study implementation due to a lapse in operations when the program was being taken over by another organization from the IRC.

During the peak lean season in July 2017, the study area reported a global acute malnutrition (GAM) prevalence of 17.7% [11]. Four out of eight *payams*, an administrative unit in South Sudan, in the county were purposively selected, focusing on areas that were accessible during the rainy season. As part of the existing iCCM program, CBDs were already trained to treat children under five in their communities for uncomplicated pneumonia, diarrhea, and malaria. They had also been instructed to refer children who present with medical complications to the nearest health facility, as well as children who meet the MUAC cutoff for SAM (<115 mm) or moderate acute malnutrition (MAM) (115 to <125 mm) to the nearest static clinics running an Outpatient Therapeutic Program (OTP, where SAM children are treated) or Targeted Supplementary Feeding Program (TSFP, where MAM children are treated) respectively.

Of the 397 iCCM CBDs active in the four selected *payams*, CBDs with the following characteristics were excluded from eligibility for this study (one person may contribute to multiple categories): male (n = 1), received any education (n = 2), lives within 5 km of the nearest OTP clinic (n = 171), CBD’s house was not accessible during rainy season (n = 9), participated in previous training or field testing of the protocol and tools (n = 19), and had travel time greater than 60 minutes to the farthest household in the catchment area (n = 1). Also, to assure a sufficient volume of SAM cases, we only included CBDs who reported serving 35 (the median value among all CBDs) or more households. This left 106 CBDs eligible across the four selected *payams*.

Out of those CBDs who met the inclusion criteria, fifteen CBDs per *payam* were randomly selected for a six-day training. The research staff obtained informed consent from CBDs to collect sociodemographic data and data on their performance following the training. Based on a performance assessment conducted at the end of the training, 11 highest performing CBDs from each *payam* who met a pre-determined minimum performance score of 80% were then deployed to deliver SAM treatment in the community. More details on the composition and the results of the performance score are described elsewhere [9]. The total number of CBDs deployed for the pilot was based on limitations of supervisory capacity of the Research Officers.

The CBDs received an additional incentive of 25 USD per month from the project to compensate them for opportunity costs they faced to provide SAM treatment in their communities. Four Research Officers hired for the project supervised the CBDs through biweekly visits. During the supervision visits, the Research Officers observed the CBDs providing SAM treatment. Based on their observations, they filled out a checklist to assess the CBDs’ performance. The Research Officers provided feedback to the CBDs during each supervision.

CBD supervisors, a pre-existing cadre from the iCCM program, were tasked with the distribution of ready-to-use therapeutic foods (RUTF) from centralized distribution points to the homes of the CBDs. Every month, IRC staff prepositioned RUTF at centralized storage facilities in each administrative unit (*payam)* the IRC operated the study in. Every week, the CBD supervisors retrieved RUTF at the storage facility closest to the CBD homes and replenished the RUTF stored at the CBD level. This way, considering the high value of RUTF in food-insecure South Sudan, the quantity of RUTF stored at the CBD home was kept to a minimum. They received an incentive of 50 USD, and an additional 15 USD for those who requested financial support for transporting the RUTF.

In addition to the incentives mentioned above, the following project inputs were provided: equipment (locked metallic boxes for RUTF storage at CBD homes and at centralized storage facilities, one set of treatment tools for each CBD), drugs (RUTF, amoxicillin, albendazole), and human resource (four Research Officers for supervision and data collection).

After conducting community sensitization meetings informing community members about the availability of malnutrition treatment from CBDs, CBDs passively identified cases among children who arrived at the CBDs’ home for iCCM and/or malnutrition treatment. They measured the child’s MUAC using the modified MUAC tape with the following color bands: dark red (<90 mm), red (90 to <102.5 mm), pink (102.5 to <115 mm), yellow (115 to <125 mm), and green (≥125 mm) (**Figure 1**). Children were eligible for treatment if their MUAC was in the red or the pink zones (90 to <115 mm), and if they had no medical complications, a good appetite, and weighed more than 4 kg. Children who had MUAC in the dark red zone (<90 mm), failed the appetite test, or weighed less than 4 kg were immediately referred to the OTP as a danger sign out of concern of severity.

The treatment protocol consisted of danger sign screening, MUAC measurement, administration of an appetite test, weight measurement, determination of daily and weekly RUTF dosage based on weight, drug provision (Amoxicillin on week 1, Albendazole on week 2), and counselling. (Full description available in Appendix S1 of the **Online Supplementary Document**.) Caregivers were instructed to return on a weekly basis for up to 16 weeks, until reaching a treatment outcome: recovered (two consecutive weeks with MUAC≥125 mm, or the green zone on the MUAC tape), default (three consecutive missed visits), non-response (had not recovered by 16 weeks), death, or referred. Referrals were made if the child presented with clinical danger signs or MUAC dropped below 90 mm. Also, without numeracy, CBDs are unable to differentiate slow regression, static, or slow progression unless MUAC values cross MUAC tape color bands. Thus, we also added a conservative protocol to refer children who stayed in the same color zone for four consecutive weeks. The sample size calculation for how many incident SAM cases to enroll in the study was based on a recovery rate of 75%, precision of 10%, alpha of 5%, and loss-to-follow-up of 10%, resulting in a sample size of 80 children per *payam*, for a total of 320 children. CBDs were asked to stop enrolling new children after the study met the required sample size.

The unreliable phone network made it difficult for the study staff to guarantee presence during the enrollment visit to administer the informed consent process. Thus, in consultation with the IRC Institutional Review Board, a two-stage consent process was developed. The CBDs were asked to receive oral consent from the caregiver for their eligible child to receive treatment from the CBD, then the study staff followed up at the caregivers’ homes to receive oral informed consent to participate in the research study and to collect sociodemographic data on the child and the household.

The study was approved by the Research Ethics Committee of the South Sudan Ministry of Health and the IRC Institutional Review Board. Informed oral consent was obtained from participating CBDs and caregivers.

### Data collection

At the weekly treatment visits conducted at the CBDs’ homes, the CBD recorded on their patient registers the MUAC color of the child and the daily dosage of RUTF sachets the child was provided. The Research Officers transposed the data from the patient registers to their own data collection forms on a weekly basis during supervision visits, until the child reached a treatment outcome. The total number of weeks in treatment and treatment outcome were captured, disaggregated by child.

Additionally, for descriptive purposes, a retrospective data audit was conducted at the static OTPs run by the IRC in each *payam*. Data on MUAC at admission, treatment outcome, and number of weeks in treatment were collected for all children who were admitted for treatment at the four static clinics between March and April 2017, the same admission period as our study.

### Data analysis

Child characteristics, treatment outcomes, and total number of weeks in treatment were summarized, with treatment outcomes examined both as recovery from SAM to MAM (two consecutive weeks in the yellow MUAC zone, or 115 to <125 mm) as well as SAM to full recovery (two consecutive weeks in the green MUAC zone, or ≥125 mm), and stratified by whether the child started treatment in the red (9 to <102.5 mm) or pink (102.5 to <115 mm) MUAC zone. The recovery rate was defined as percent recovered, excluding children who were referred from the denominator, per standard nutrition metrics, but data are also presented with recovery as proportion of total enrolled children (including referrals in the denominator). Univariate and multivariate log-binomial regression models, controlling for clustering at the CBD level, were run with recovery as the outcome variable of interest, and with independent variables of child age, child sex, MUAC color at admission, having not received malnutrition treatment in last four months, number of under-five children in the household, and the last available CBD performance score from the biweekly performance assessments by study staff (described in detail in separate publication [9]). For comparison, OTP data on treatment outcomes and weeks in treatment were summarized, both in aggregate and stratified by MUAC color at admission. Stata (version 13) was used for data analysis.

## RESULTS

### Description of participants

The characteristics of the CBDs are available in **Table 1**.

**Table 1:** Characteristics of community-based distributors (CBD)

Characteristics	N (%)
**Age (years):**	
18-24	7 (15.9)
25-34	15 (34.1)
35-44	9 (20.5)
45-54	8 (18.2)
55+	5 (11.4)
**Ability to read:**	
Yes	1 (2.3)
No	43 (97.7)
**Number of pregnancies:**	
0	1 (2.3)
1-3	7 (15.9)
4-6	23 (52.3)
7+	13 (29.6)
**Religion:**	
Christian	31 (70.5)
Traditional	13 (29.6)
**Occupation:**	
None	2 (4.6)
Farmer	38 (86.4)
Commerce	4 (9.1)
**Number of years working as CBD:**	
<1	0 (0)
1-2	1 (2.3)
3-4	25 (56.8)
5-6	18 (40.9)
**Estimated number of households served:**	
Mean	46.2
Median	44
IQR	40-50.5
Range	30-70

CBD – community-based distributor, IQR – interquartile range

Of the 44 CBDs deployed, a total of 40 CBDs treated at least one child in the community. A total of 314 children were enrolled. No child was enrolled on oedema. Research Officers identified four children as receiving treatment from both a CBD and an OTP site, and when asked to select one, all four chose to withdraw from the study and to continue care from the OTP site for reason of proximity. Two children were reenrolled following discharge and their second records were excluded from the analysis to avoid inclusion of the same child twice, leaving a total of 308 children treated with an eligible outcome. Their characteristics are available in **Table 2**. Each CBD treated a median of 7 children (mean = 7, interquartile range (IQR) = 6-9, range = 1-15) over the course of the study. As a proxy for workload, the maximum number of children treated by each CBD in a single week was a median of 7 children (mean = 7, IQR = 6-8, range = 1-12).

**Table 2:** Characteristics of eligible uncomplicated severe acute malnutrition cases (n = 308 children)

Characteristic	n (%)
**Sex:**	
Male	140 (45.5)
Female	168 (54.6)
**Child age (in months):**	
Mean	21.1
Median	24
IQR	12-24
Range	6-59
**Received malnutrition treatment in last 4 months:**	
Yes	47 (15.3)
No	260 (84.4)
Don’t know	1 (0.3)
**Number of under-fives in the household:**	
Mean	2.2
Median	2
IQR	2-3
Range	1-6
**Household size:**	
Mean	6.9
Median	7
IQR	6-8
Range	3-13
**Religion:**	
Christian	239 (77.6)
Traditional	68 (22.1)
Don’t know / missing	1 (0.3)
Maternal education:	
No education	299 (97.1)
Literacy course	4 (1.3)
Primary education	4 (1.3)
Don’t know / missing	1 (0.3)
**Paternal education:**	
No education	282 (91.6)
Literacy course	2 (0.6)
Primary education	14 (4.5)
Secondary education and up	6 (1.9)
Don’t know/missing	4 (1.3)
**Mid-upper arm circumference at admission:**	
Red zone (90-102.5 mm)	90 (29.2)
Pink zone (102.5-115 mm)	218 (70.8)

IQR – interquartile range

*Treatment outcome.* The recovery rate from SAM to MAM, excluding referrals from the denominator, was 91% (95% confidence interval (CI) = 88%-95%). The remaining 9% (95% CI = 5%-12%) defaulted. The treatment outcomes are available in **Table 3**. The median length of treatment among those who recovered to MAM was five weeks (mean = 5, IQR = 4-6 weeks, range = 3-15).

**Table 3:** Treatment outcomes of children treated by community-based distributors (CBD), accounting for clustering at CBD level

	Recovery from SAM to MAM (two consecutive weeks in yellow MUAC zone, 115 to <125 mm)			Recovery from SAM to full recovery (two consecutive weeks in green MUAC zone, ≥125 mm)		
	**n**	%, out of those discharged* (95% CI)	**%, out of total exits† (95% CI)**	**n**	%, out of those discharged* (95% CI)	**%, out of total exits† (95% CI)**
Recovered	222	91.3 (86.6-94.5)	71.8 (64.7-77.8)	147	75.4 (68.3-81.3)	47.8 (40.4-55.2)
Defaulted	21	8.8 (5.5-13.4)	6.8 (4.2-10.9)	30	15.4 (10.8-21.4)	9.7 (6.6-14.2)
Non-response	0	0	0	18	9.2 (4.8-16.9)	5.8 (3.1-10.7)
Death	0	0	0	0	0	0
Referred	65	Not applicable	21.4 (15.1-29.6)	113	Not applicable	36.7 (28.9-45.2)

SAM – severe acute malnutrition, MAM – moderate acute malnutrition, MUAC – mid-upper arm circumference, CI – confidence interval

*Per standard nutrition program definitions, discharge excludes referrals and exits.

†Includes referrals in the denominator.

The recovery rate from SAM to full recovery was 75% (95% CI = 69%-81%). The median length of treatment among those who recovered fully was 8 weeks (mean = 9, IQR = 6-11, range = 3-16). 15% (95% CI = 11%-21%) defaulted and 9% (95% CI = 5%-13%) did not respond after 16 weeks of treatment. Median time to default was 5 weeks (mean = 6, IQR = 4-7, range = 3-13). 94% of the 113 children referred between SAM and full recovery were for a protocol safeguard that we had instituted for children that had the same MUAC color for four consecutive weeks; however, it is worth noting here that we cannot differentiate these cases as those that were slowly regressing, static, or slowly progressing. Reasons for default and referral are available in **Table 4** and **Table 5**, respectively. No deaths were reported throughout the study.

**Table 4:** Reported reasons for default

	Outcome from SAM to MAM (two consecutive weeks ≥115 mm) (n=21)		Outcome from SAM to full recovery (two consecutive weeks ≥125 mm) (n=30)*	
	**n**	**%**	**n**	%
Child moved away	12	57.1	12	40.0
Caregiver did not have time to bring child	3	14.3	10	33.3
Child went to OTP site	3	14.3	3	10.0
Admitted at health facility	0	0	1	3.3
Don’t know/missing	3	14.3	4	13.3

SAM – severe acute malnutrition, MAM – moderate acute malnutrition, OTP – outpatient therapeutic program

*Includes the values of n=21 from left column.

**Table 5:** Reported reasons for referral

	Outcome from SAM to MAM (two consecutive weeks ≥115 mm) (n=65)		Outcome from SAM to full recovery (two consecutive weeks ≥125 mm) (n=113)*	
**n**	**%**	**n**	**%**
**4 consecutive MUAC colors:**				
-Red (90 to <102.5 mm)	8	12.3	8	7.1
-Pink (102.5 to <115 mm)	54	83.1	54	47.8
-Yellow (115 mm to <125 mm)	–	–	44	38.9
**iCCM specific danger signs**	1	1.5	2	1.8
**MUAC measurement below admissions MUAC**	2	3.1	3	1.8
**Don’t know/missing**	–	–	2	2.7

SAM – severe acute malnutrition, MAM – moderate acute malnutrition, MUAC – mid-upper arm circumference, iCCM – integrated community case management (of childhood illness)

*Includes the values of n=65 from left column.

The treatment outcomes were stratified by the MUAC at admission (**Table 6**). The recovery rate to MAM for MUAC 90 to <102.5 mm (red MUAC band) at admission was 88% (79%-93%) and for MUAC 102.5 to <115 mm (pink MUAC band) at admission was 93% (88%-96%). The recovery rate to full recovery for MUAC 90 to <102.5 mm at admission was 71% (61%-80%) and for MUAC 102.5 to <115 mm at admission was 78% (68%-85%). The median length of stay for MUAC 90 to <102.5 mm at admission to full recovery was 9 weeks (mean = 10, IQR = 7-13 weeks, range = 4-16) and for MUAC 102.5 to <115 mm at admission was 7 weeks (mean = 8, IQR = 6-9 weeks, range = 3-16).

**Table 6:** Treatment outcome, stratified by MUAC at admission, excluding referrals

	MUAC 90-<102.5 mm at admission (red MUAC)		MUAC 102.5-1.5 mm at admission (pink MUAC)	
	**n**	**% (95% CI)**	**n**	**% (95% CI)**
**Excluding referrals:**				
**Outcome from SAM to MAM (two consecutive weeks ≥115 mm)**				
Recovered	64	87.7 (78.5, 93.3)	157	92.9 (87.8, 96.0)
Defaulted	9	12.3 (6.7, 21.5)	12	7.1 (4.0, 12.2)
Non-response	0	0	0	0
Death	0	0	0	0
**Outcome from SAM to full recovery (two consecutive weeks ≥125 mm)**				
Recovered	47	71.2 (61.0, 79.7)	100	77.5 (68.4, 84.6)
Defaulted	11	16.7 (9.9, 26.8)	19	14.7 (9.3, 22.6)
Non-response	8	12.1 (5.4, 24.9)	10	7.8 (2.9, 19.2)
Death	0	0	0	0
**Including referrals:**				
Outcome from SAM to MAM (two consecutive weeks ≥115 mm):				
Recovered	64	71.1 (59.3, 80.6)	157	72.0 (63.9, 78.9)
Defaulted	9	10.0 (5.3, 17.9)	12	5.5 (3.0, 9.9)
Non-response	0	0	0	0
Death	0	0	0	0
Referrals	17	18.9 (10.2, 32.3)	49	22.5 (15.1, 32.1)
**Outcome from SAM to full recovery (two consecutive weeks ≥125 mm)**				
Recovered	47	52.2 (39.9, 64.3)	100	45.9 (38.1, 53.8)
Defaulted	11	12.2 (7.1, 20.1)	19	8.7 (5.0, 14.2)
Non-response	8	8.9 (4.1, 18.3)	10	4.6 (1.7, 11.7)
Death	0	0	0	0
Referrals	24	26.7 (15.7, 41.5)	89	40.8 (32.1, 50.2)

SAM – severe acute malnutrition, MAM – moderate acute malnutrition, MUAC – mid-upper arm circumference

### Predictors of recovery

The child had 7% increased chance of recovery (adjusted Risk Ratio (aRR) = 1.07, 95% CI = 1.02-1.13) for every year older the child was. Those children who had not received any malnutrition treatment in the last four months had an 18% decreased chance of recovery (aRR = 0.82, 95% CI = 0.72-0.93) (**Table 7**). When including referrals in the reference group, there were no statistically significant predictors of recovery.

**Table 7:** Risk ratios (RR) of recovery, accounting for clustering at CBD level

	Recovery (excluding referrals in reference group*), (n=195)		Recovery (including referrals in reference group*), (n=308)	
	**Unadjusted RR**	**Adjusted RR**	**Unadjusted RR**	**Adjusted RR**
Age of child (in years, 0-5)	1.06 (1.01, 1.11)	1.06 (0.96, 1.17)	1.06 (0.96, 1.17)	1.07 (1.02, 1.13)
Sex of child	0.90 (0.77, 1.05)	0.99 (0.79, 1.23)	0.99 (0.79, 1.23)	0.88 (0.77, 1.01)
MUAC color at admission, Ref: red	1.12 (0.96, 1.31)	0.92 (0.71, 1.18)	0.92 (0.71, 1.18)	1.15 (0.98, 1.34)
Has not received malnutrition treatment in last 4 months	0.81 (0.71, 0.93)	0.77 (0.62, 0.96)	0.77 (0.62, 0.96)	0.82 (0.72, 0.93)
Number of under-five children in the house	1.09 (1.00, 1.18)	1.11 (0.93, 1.32)	1.11 (0.93, 1.32)	1.10 (1.00, 1.21)
Final performance score, in 10-percentage point increments	1.01 (0.90, 1.14)	1.13 (0.92, 1.38)	1.13 (0.92, 1.38)	1.01 (0.94, 1.09)

CBD – community-based distributor

*Reference group includes defaulters and non-responders. No deaths were reported in the study. The unadjusted risk ratio (RR) is univariate regression analysis, and the adjusted RR is multivariate regression analysis including all the independent variables listed here.

### Descriptive data from nearest OTPs

For SAM cases admitted at the OTPs in the study *payams* for the same admission period (March-April 2017) (**Table 8**), there was a lower proportion of children admitted in the MUAC red zone (90 to <102.5 mm) vs the pink zone (102.5 mm to <115 mm) at the OTP (5%) compared to with the CBD (29%), suggesting that children accessing care from CBDs were more severely malnourished, as assessed by MUAC. For the OTPs, the children with MUAC 90-102.5 mm at admission had a MAM recovery rate (defined as two consecutive visits with MUAC≥115 mm) of 36% (n = 5 recovered, n = 2 default, n = 7 non-response, n = 0 died, with additional 3 children referred) and children with MUAC 102.5-115 mm at admission had a MAM recovery rate of 82% (n = 236 recovered, n = 36 default, n = 15 non-response, n = 0 died, with additional 16 children referred). Of the 241 children who recovered from SAM to MAM, only 108 (45%) children had admission records at the TSFP. Only two children with MUAC 90 to <102.5 mm at admission had records at the TSFP and both recovered, and children with MUAC 102.5 to <115 mm at admission had a full recovery rate (defined as two consecutive visits with MUAC≥125 mm) of 71% (n = 63 recovered, n = 17 default, n = 8 non-response, with additional 19 referred). A direct statistical comparison of recovery rates between CBD and OTP treatment was not made, due to expected differences in sociodemographic characteristics as well as differences in treatment protocol. For instance, children treated by CBDs had a lower MUAC at admission and are suspected to have lower socioeconomic status. Additionally, severely malnourished children who progressed to MAM under CBD treatment received more and higher quality caloric content than those treated at the TSFP, as the former were provided RUTF by weight until full recovery (ranging from 2-5 RUTF sachets per day, on a weekly basis) and the latter received two bags of fortified blended foods every two weeks.

**Table 8:** Treatment outcomes of cases admitted at four outpatient therapeutic programs in study area, March-April 2017

	Outcome from SAM to MAM (two consecutive weeks ≥115 mm)			Outcome from SAM to full recovery (two consecutive weeks ≥125 mm)*		
	**n**	**%, out of those discharged**† **(95% CI)**	**%, out of total exits**‡ **(95% CI)**	**n**	**%, out of those discharged**† **(95% CI)**	**%, out of total exits**‡ **(95% CI)**
Recovered	241	80.1 (75.1, 84.2)	75.3 (70.3, 80.0)	64	71.9 (61.5, 80.4)	59.3 (49.6, 68.2)
Defaulted	38	12.6 (9.3, 16.9)	11.9 (8.7, 15.9)	17	19.1 (12.1, 28.8)	15.7 (9.9, 24.0)
Non-response	22	7.3 (4.8, 10.9)	6.9 (4.6, 10.2)	8	9.0 (4.5, 17.2)	7.4 (3.7, 14.2)
Death	0	0	0	0	0	0
Referred	19	Not applicable	5.9 (3.8, 9.1)	19	Not applicable	17.6 (11.4, 26.1)

SAM – severe acute malnutrition, MAM –moderate acute malnutrition, CI – confidence interval

*Data taken from both outpatient therapeutic program and therapeutic supplemental feeding program records.

†Per standard nutrition program definitions, discharge excludes referrals and exits,

‡Includes referrals in the denominator.

## DISCUSSION

The CBD-delivered acute malnutrition treatment model showed high recovery rates among uncomplicated SAM cases, despite the underlying food insecurity and the low literacy of the CBD cadre. These data, combined with the high CBD performance scores for protocol adherence reported in a separate publication [9], show promise for deploying CHWs to provide acute malnutrition treatment, regardless of their literacy levels. The pilot was not powered or intended to capture longer-term health outcomes or changes in incidence or prevalence of acute malnutrition. In a low-income, conflict-affected context like South Sudan, a large proportion of under-five deaths is still driven by the preventable infectious causes treated by iCCM [12]; given the well-documented relationship between infection and malnutrition [13], a program design that treats both infection and malnutrition in the community may have potential to more effectively reduce incidence of both.

The children enrolled by the CBDs had lower MUAC at admission than those at the nearest OTPs, and a large majority of the former reported that they had not received any malnutrition treatment in the last four months. While we did not have the resources to assess change in treatment coverage attributable to this pilot, this point, along with the selection of CBDs residing more than 5 km from the nearest OTP for our study, suggests that the model may have successfully reached those who were previously not receiving care rather than replacing OTP services. There is very limited evidence from studies with designs that allow for strong causal inference on the impact on coverage [8]; a previous study conducted in Mali demonstrated a statistically significant change in coverage, comparing intervention and control (intervention arm coverage from 43.9% to 86.7%, control arm coverage from 43.8% to 41.6%, *P* < 0.05) [14]. The same study also showed cost-effectiveness [15]. Further exploration is necessary to understand the net effect of introducing a CBD-delivered malnutrition treatment model at scale, and the cost-effectiveness of such a model, taking into account collateral outcomes beyond change in coverage and treatment outcomes.

One challenge to developing a low-literacy protocol was how to monitor whether cases are stationary, slowly regressing, or slowly progressing in treatment, given that numeric MUAC values could not be captured by our CBDs. To address this, we created smaller MUAC color zones (separating the traditional MUAC tape red zone of <115 mm to red zone of 90 to <102.5 mm and pink zone of 102.5 to <115 mm) and set a safeguard for referral after four consecutive weeks in the same color band. Based on the larger than expected proportion of referrals for this reason, we will have to conduct further research to determine whether this is driven by program performance vs a protocol that was too conservative. Further exploration is needed to adjust this safeguard.

**Figure 2:**
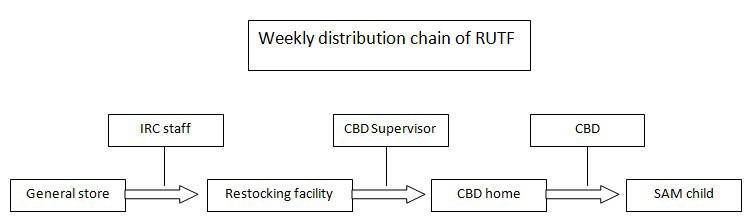
Supply chain system for the study.

Treating malnutrition in the community introduces several operational challenges in addition to those that affect facility-based programs. First, the community acceptability of CHWs treating acute malnutrition is unclear. In qualitative data collected for this pilot, community members expressed general acceptability, but also expressed suspicions that CBDs may be operating on favoritism since CBDs turned away some children from treatment. CBDs in response noted that they clearly explained to the caregivers using the MUAC tape that only children whose MUAC landed in the red or pink zone qualified for treatment from them [9]. Second, RUTF needs to be stored in the CHWs’ homes. While no major incidents were reported during the course of the study, a few CHWs expressed initial anxiety of keeping supply in their house (Unpublished data). Another challenge is that the supply chain needs to reach the CHWs’ homes (see **Figure 2** for supply chain model used). CBD supervisors, who were responsible for RUTF delivery had difficulty with moving the physical bulk of the supply, despite the fact that one CBD supervisor had no more than 3 CBDs to stock. The iCCM model seeks to provide physically proximate care for all children, but given the difference in bulk of the commodity comparing iCCM treatment (amoxicillin, ORS, ACT) to malnutrition treatment (RUTF) and the lower incidence of SAM compared to iCCM conditions, the CBD-delivered malnutrition treatment model may need to be less saturated, or in other words, one CBD may need to serve a larger number of households specifically for acute malnutrition. We also note here that the level of supervision available to the CBDs in this study was more than a standard iCCM program affords to the CBDs; iCCM CBDs receive supervision at a monthly frequency, but often less frequently due to logistical constraints, and there is a possibility that treatment outcomes will deteriorate when the nutrition program receives less supervision than what was provided in our study.

There are several limitations to the study. The original objective of the study was for iCCM and malnutrition treatment to be delivered at the same time, hence the existing iCCM CBD cadre was recruited for the study. However, the iCCM program funding transitioned from IRC to another organization in April 2017, and the subsequent organization did not start up the iCCM program operations until after September 2017. It is unclear if and how the treatment outcomes were impacted by this. Our pilot project provided the CBDs with amoxicillin and albendazole per the malnutrition treatment protocol, but the CBDs reported stock-outs in ORS and ACTs due to this pause in iCCM programming. Treatment outcomes may have been worse with a more complicated service delivery model and larger workload for the CBDs with the iCCM program operating, but it is also possible that treatment outcomes may have been better than what was seen here, with successful co-treatment of iCCM conditions. Given that this was a feasibility study, we had a small sample size of CBDs, purposively selected to ensure physical access of the research study team over the duration of the study. We cannot ascertain that the capacity of the CHWs selected and those who were not are comparable, and may see different results in a scaled program.

## CONCLUSION

The high recovery rate for SAM children enrolled in acute malnutrition treatment by low-literate CBDs shows promise that deploying CBDs to treat SAM in areas with high prevalence and low treatment access may lead to higher recovery, better continuity of care in the transition between severe and moderate acute malnutrition, and shorter treatment time. Proper adaptations of tools and protocols can empower CHW cadres with no formal education to successfully complete life-saving tasks. Key questions of scalability and cost-effectiveness remain.

## Additional material

Online Supplementary Document
