# Supplementary material for: Severe acute malnutrition treatment delivered by low-literate community health workers in South Sudan: A prospective cohort study
Source: J Glob Health. 2020 Jun 11;10(1):010421. doi: 10.7189/jogh.10.010421 (PMC7295452; doi:10.7189/jogh.10.010421)
Supplement: Online Supplementary Document [file jogh-10-010421-s001.pdf]

Simplified Protocol for the Treatment of Uncomplicated Severe Acute Malnutrition Integrated into iCCM

Simplified Tools and Training Guidelines

Developed with support from the Eleanor Crook Foundation

Introduction

This is a guidebook for training front line low-literate community health workers (CHWs) in the use of the simplified tools and protocol for treating severe acute malnutrition (SAM) and uncomplicated severe acute malnutrition. Step-by-step instructions and images show how to use each tool and how to follow the simplified protocol to treat children suffering from uncomplicated severe acute malnutrition.

Part 1 describes the simplified tools and how to use them

> **MUAC Tape:** a modified mid-upper arm circumference (MUAC) tape with additional color zones that can be used for admission, monitoring and discharge purposes
>
> **RUTF Dosage Scale:** a dot system used with the standard Salter scale to help those who cannot read numbers determine the correct daily dosage of Ready-to-Use Therapeutic Food (RUTF). Each cluster of dots represents the number of RUTF sachets to be given daily based on the child's weight
>
> **RUTF Dosage Calculator:** a device that has individual pockets for each day of the week to help CHWs count and give out the correct weekly (7 day) dosage of RUTF
>
> **Patient Register:** a register that does not require the ability to read in order to use, and captures all the important patient details and weekly monitoring readings over four months.
>
> **Key messages for how to give RUTF at home:** a flipchart that demonstrates the key steps for how to give RUTF at home. This tool aids CHWs in explaining the RUTF feeding process to caregivers.

Part 2 describes essential procedures (non-tool related) for diagnosing and treating severe acute malnutrition

Part 3 describes the full simplified SAM treatment protocol

Part 4 describes the iCCM + SAM treatment protocol

Table of Contents

Acronyms 7

Part 1: Simplified Tools

1.1 MUAC Tape 11

1.2 RUTF Dosage Scale..................................19

1.3 RUTF Dosage Calculator...........................27

1.4 Patient Register.......................................31

1.5 Key RUTF Feeding Messages.......................47

Part 2: Essential Procedures

Checking for Bilateral Pitting Oedema.............53

Appetite Test..55

Danger Signs...59

Part 3: Simplified SAM Treatment Protocol

3.1 Week 1..69

3.2 Week 2...79

3.3 Week 3-16 85

3.4 Discharge Criteria 91

Part 4: iCCM + SAM Treatment Protocol

Malaria + SAM...100

Pneumonia + SAM..102

Diarrhea + SAM 104

Acronyms

CHW
Community Health Worker

iCCM
Integrated Community Case Management

ID
Identification

MUAC
Mid-Upper Arm Circumference

OTP
Outpatient Therapeutic Program

PHCC
Primary Health Care Center

PHCU
Primary Health Care Unit

RUTF
Ready-to-use Therapeutic Food

SAM
Severe Acute Malnutrition

TSFP
Targeted Supplementary Feeding Program

Part 1:
Simplified Tools

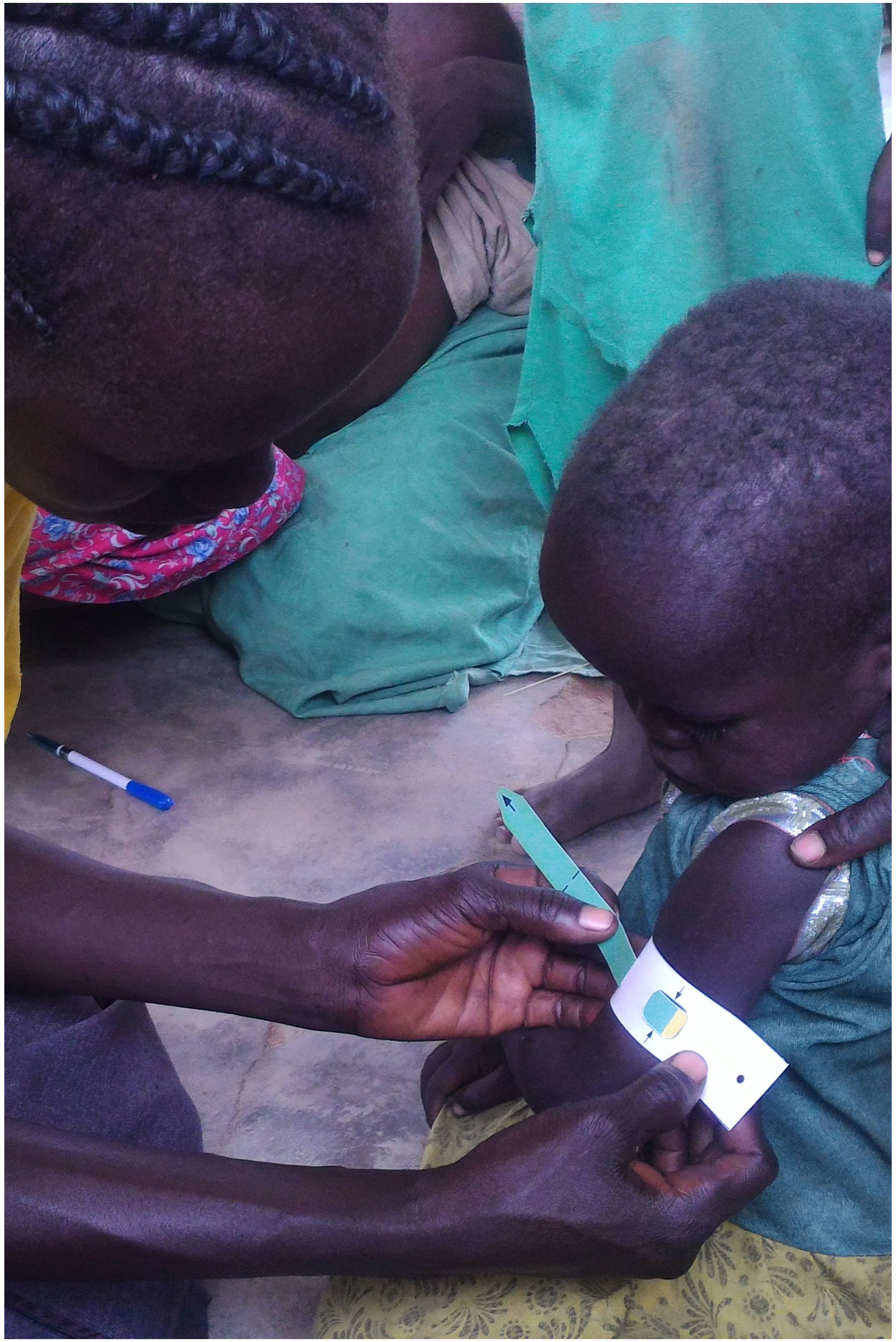

1.1
Mid-Upper Arm Circumference Tape (MUAC Tape)

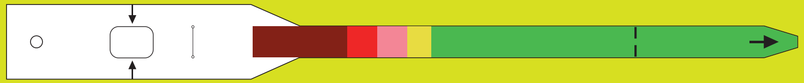

MUAC Tape: How to use

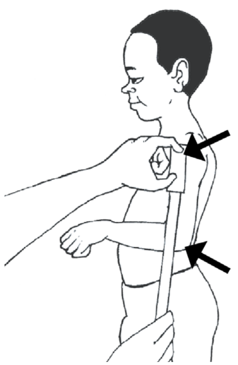

1. Locate the midpoint of the child's upper arm (bicep area) using a string. (Do not bend the MUAC tape in half to find the midpoint)

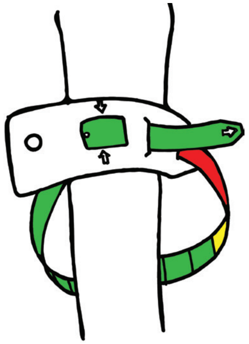

2. Wrap tape around the midpoint of the child's upper arm.

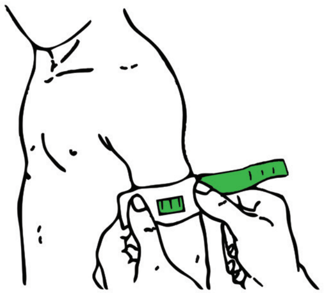

3. Slide the pointed end of the green zone (end of the tape) through the narrow slit in the white part of the tape.

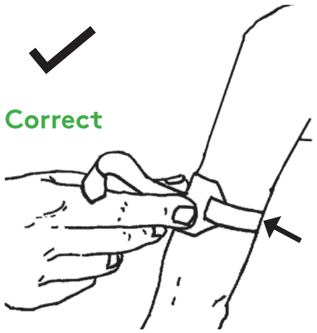

4. Pull the tape until it fits snugly against the child's arm, neither too loosely nor too tightly.

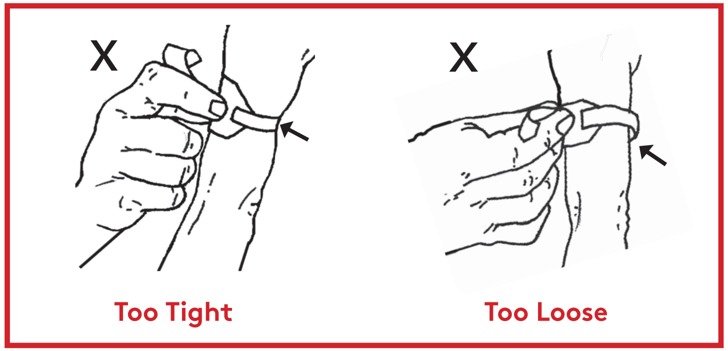

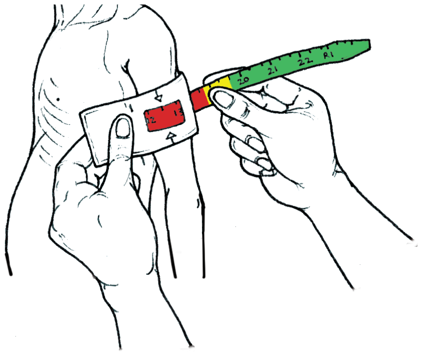

5. While holding down the white end of the MUAC, read the color between the two arrows. This shows child's status.

Suggested Training Activities

1. Each CHW should take MUAC on her neighbor while explaining the key steps (even if the MUAC doesn't wrap fully around the arm)
2. Color identification: Show CHWs the cards with the different MUAC colors and have them practice identifying the colors, first in order, then out of order. Ask which colors are different from the MUAC they used in the past.
3. Prepare several bamboo rods of different diameters and number each one. Two trainers should each take the MUAC of each rod and record the color. Each CHW should take several MUAC readings of different size bamboo rods. Trainers should check the results against their measurements and help the CHWs to correct their errors.
4. Prepare a practice session where CHWs will measure and record the color of MUAC on children from the OTP. Check for common errors including identifying the midpoint, having the tape too loose and having the tape too tight.

Materials Needed

1. New MUAC tapes
2. Cards in the five MUAC colors
3. Bamboo rods of different diameters
4. Pens

MUAC Tape: What it means

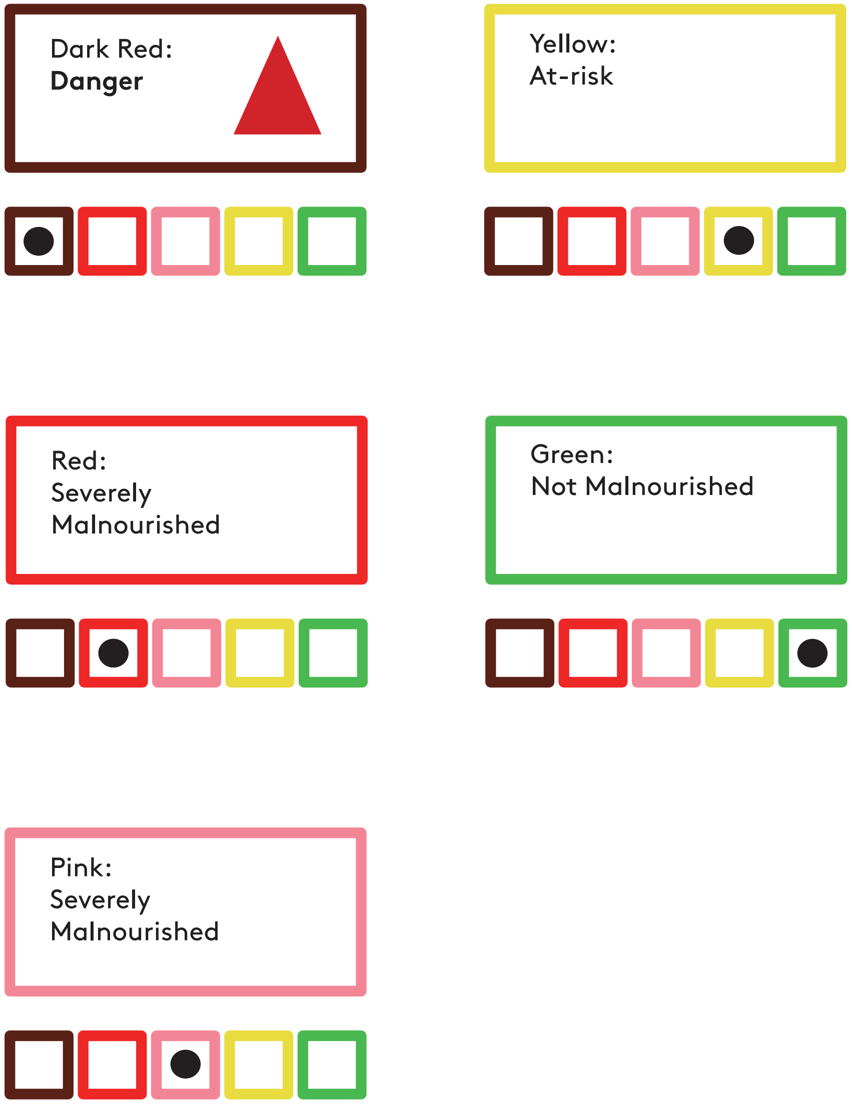

MUAC Tape: How to record

1. Tick the box with the same color as the MUAC reading in the patient register.

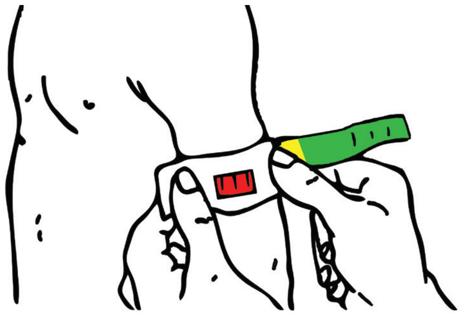

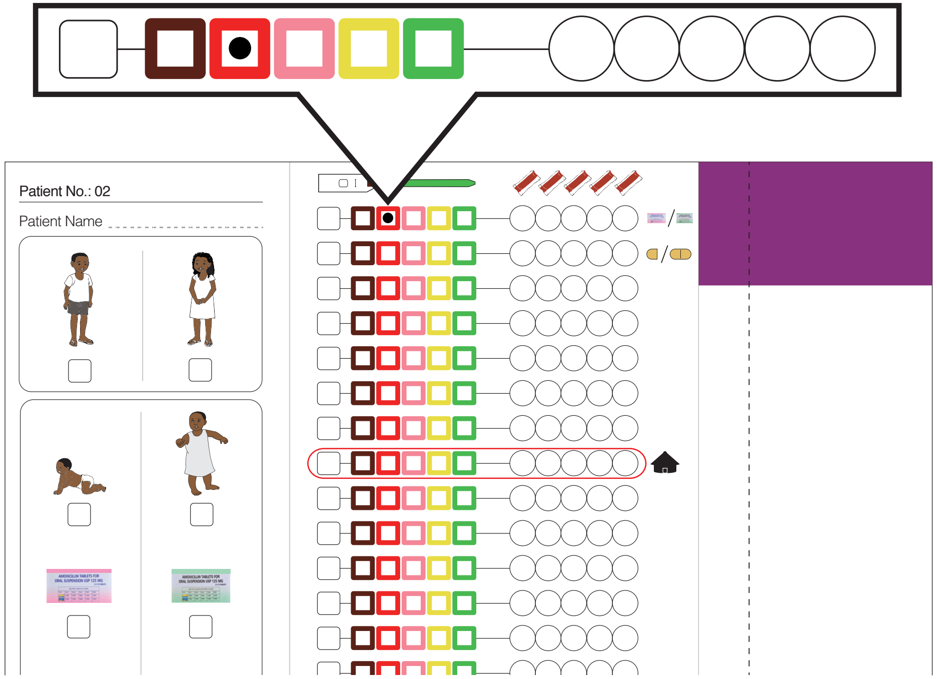

Suggested Training Activities

1. Trainers show cards with different MUAC colors and CHWs practice marking the correct color on the register.
2. CHWs practice measuring bamboo rods and recording the color on the register.
3. CHWs measure children from the OTP and record their measurement on the register.

Materials Needed

1. New MUAC tapes
2. Cards in the five MUAC colors
3. Bamboo rods of different diameters
4. Individual color copies of the register sheet
5. Pens

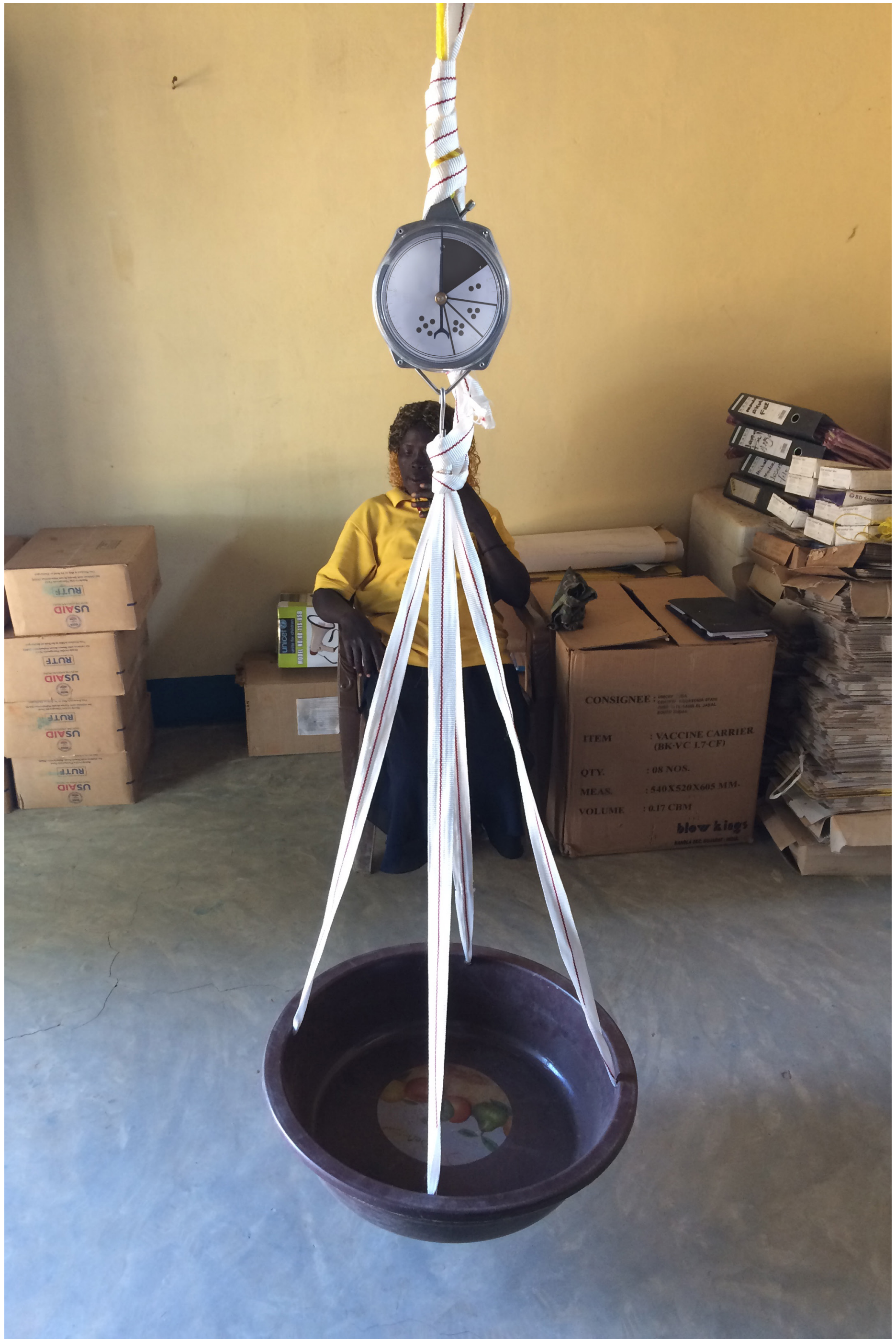

1.2 Ready-to-Use Therapeutic Food (RUTF) Dosage Scale

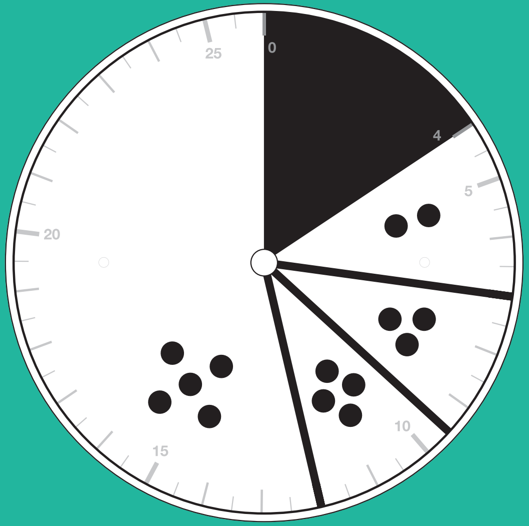

Dosage Scale: How to use

Setting up the scale:

1 . Find a sturdy branch or beam to hang the scale.

2 . Ensure that the scale and basin are tied securely and the basin is hanging low to the ground.

3 . Set the scale to 0 (the arrow should point straight at the zero like the below image).

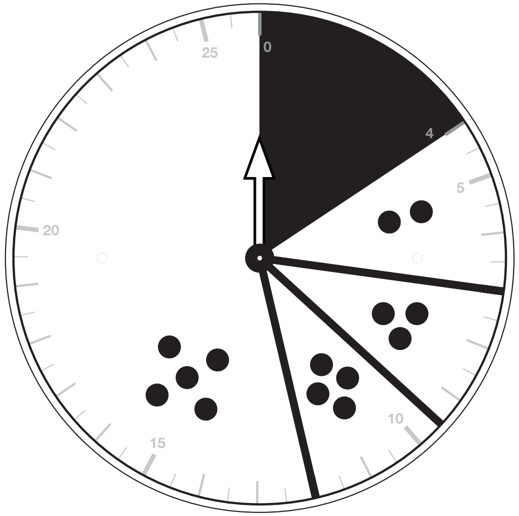

Suggested Training Activities

1. Have CHWs identify appropriate locations in the training area to hang a scale and practice hanging up the scale and basin.
2. Set the scale to less than 0 or greater than 0 and have the CHWs adjust it to the right location.

Materials Needed

1. Salter scales
2. Basins
3. Rope

Dosage Scale: How to use

Weighing the Child:

1 . The child should be undressed as much as possible.

2. Put the child into the basin and weigh the child using the hanging scale.

3. See the zone the scale's arrow stops in and count the number of dots in it.

4 . The number of dots is the number of RUTF sachets the child is to eat each day.

5. If the arrow falls on the black line, round up to the next zone.

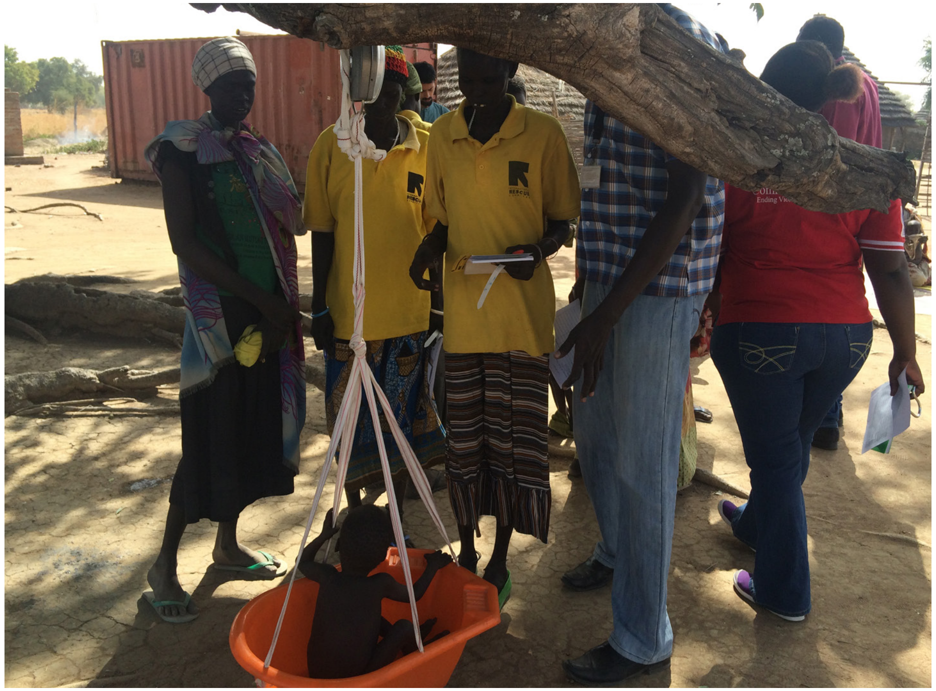

Dosage Scale: What it means

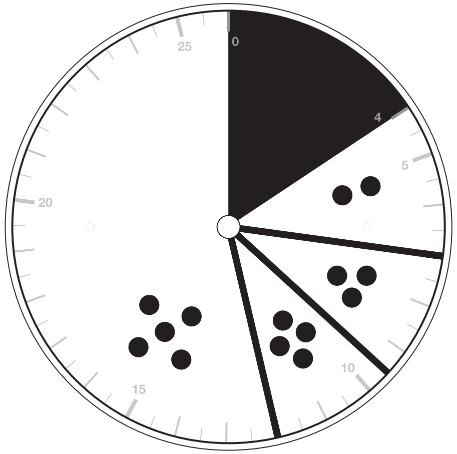

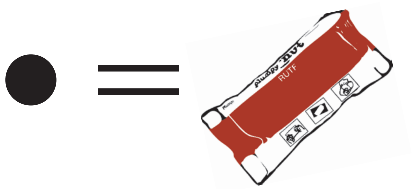

Black area
Refer immediately

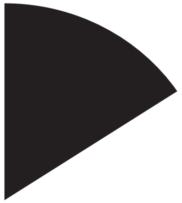

=

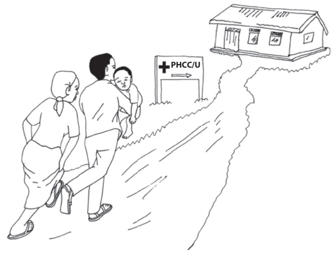

2 dots = 2 sachets of RUTF per day

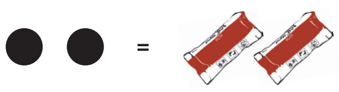

4 dots = 4 sachets of RUTF per day

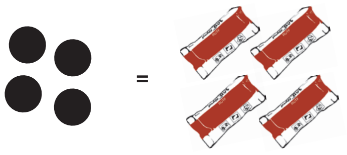

3 dots = 3 sachets of RUTF per day

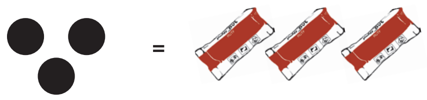

5 dots = 5 sachets of RUTF per day

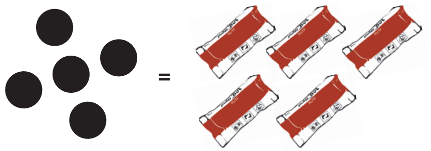

Suggested Training Activities

1. Show the CHWs the cards with the different numbers of dots on them and have them practice counting.
2. Prepare bags with various weights for CHWs to practice weighing and identifying number of sachet (including weights on the border regions).
3. If possible have CHWs practice weighing children from the OTP.

Materials Needed

1. Cards with different numbers of dots on them
2. Salter scale, basin, rope
3. Bags with different weights
4. Individual color copies of the register sheet

Dosage Scale: How to record

1. Make one tick mark for each dot (sachet of RUTF) in the correct space on the register like the example below.

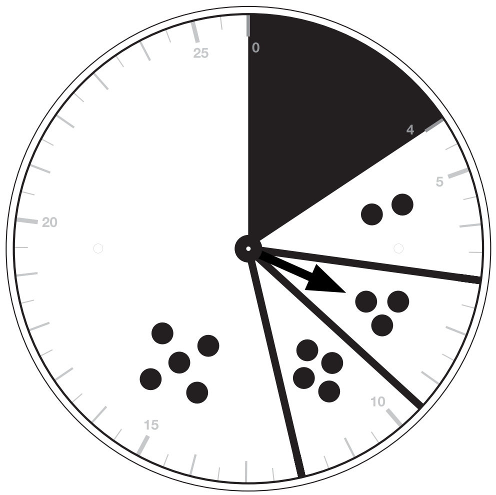

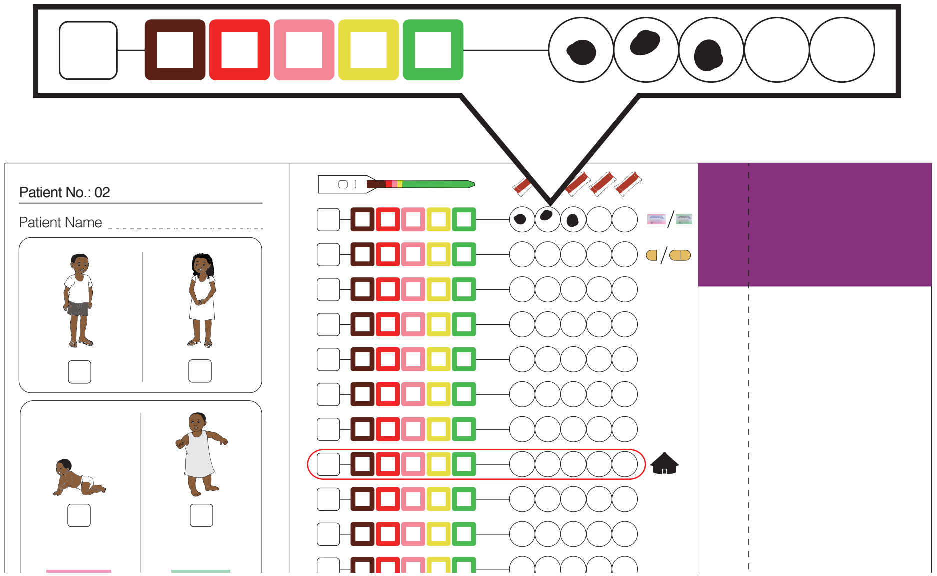

Suggested Training Activities

1. Show CHWs the cards with different numbers of dots on them and have them mark the corresponding place on the register.
2. Prepare bags with various weights for CHWs to weigh to practice identifying and recording the number of sachets.
3. Have CHWs practice weighing children from the OTP and recording the corresponding number of sachets.

Materials Needed

1. Cards with 2-5 dots on them and one card that is black
2. Salter scale, basin, rope
3. Bags with different weights
4. Individual color copies of the register sheet

Dosage Calculator: How to use

1. Place the number of RUTF sachets per day on each square in the calculator.

2. Explain to the caregiver that each square represents the number of sachets the child should eat each day.

3. When all seven days have been counted out, double check, then transfer the weekly supply to a carrying bag for the caregiver.

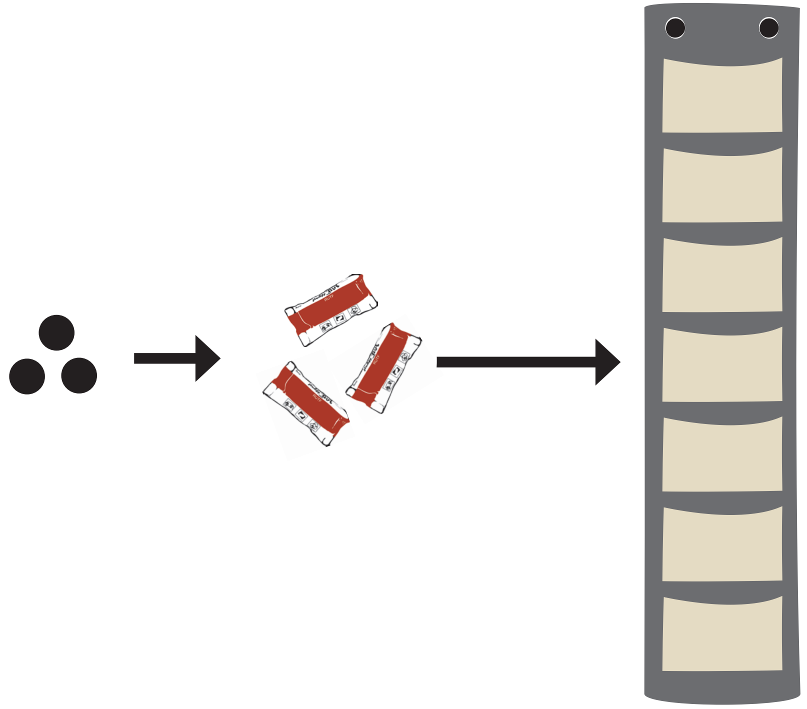

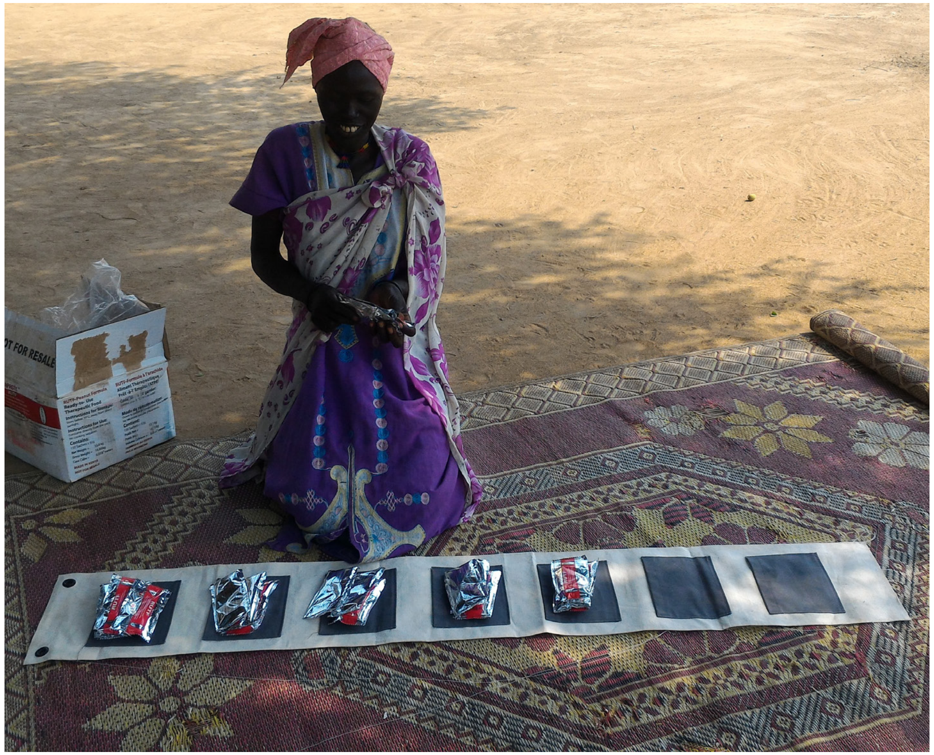

Suggested Training Activities

1. Practice counting days 1-7 using the pockets on the dosage calculator.
2. Show CHWs the cards with the different number of dots on them and have them practice counting out a full week of RUTF sachets using the calculator.
3. Prepare bags with various weights for CHWs to weigh to practice identifying and preparing the number of sachets to distribute.
4. Have CHWs practice weighing children from the OTP, preparing the corresponding number of sachets, and recording on the individual register sheets.

Materials Needed

1. Dosage calculator
2. Cards with 2-5 dots on them and one card that is black
3. Salter scale, basin, rope
4. Bags with different weights
5. Individual color copies of the register sheet
6. Pens

1.3
Patient Register

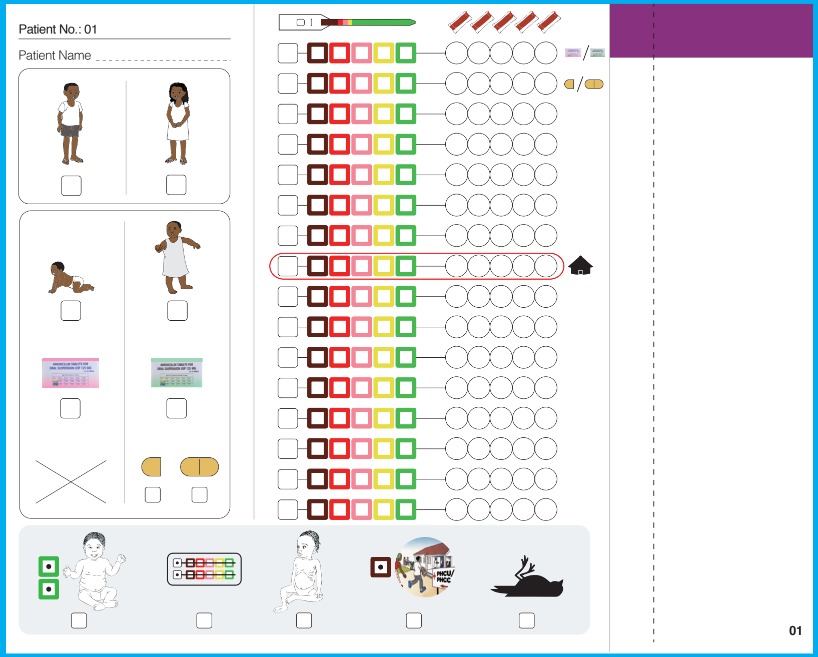

Patient Register: Sex

1. This is filled in when the child is admitted for the first time.

2. Ask the caregiver the sex of the child.

3. Mark the box below the image that matches to the child's sex.

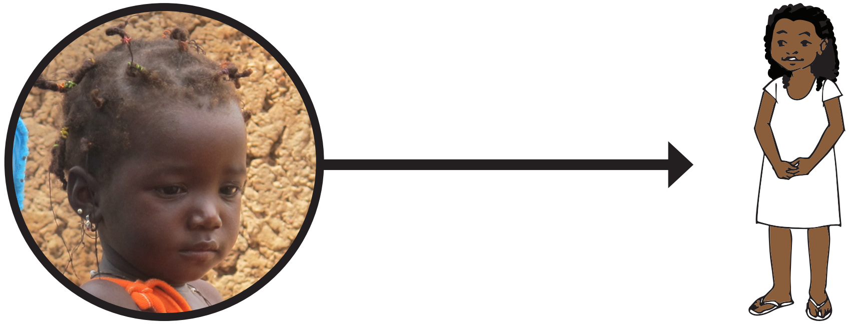

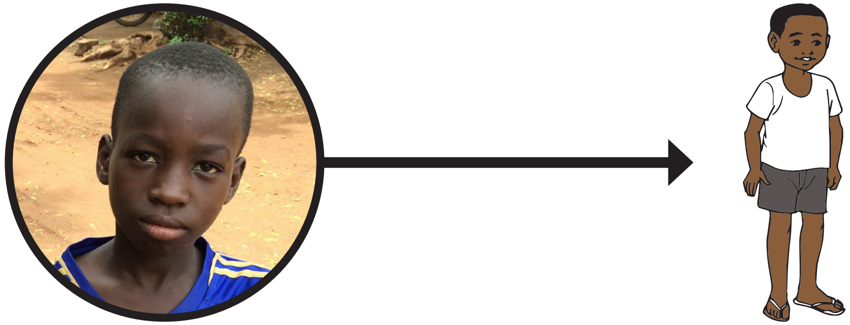

Patient Register: Age

1. This is filled in when the child is admitted for the first time.

2. Determine if the child is an infant (i.e., 6-11 months) or a toddler. (i.e., 12-59 months)

3. Mark the box below the image that matches to the child's age group. An infant is crawling. A toddler is standing.

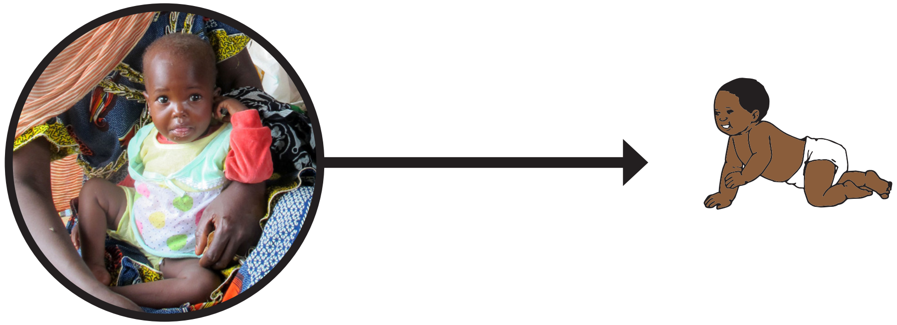

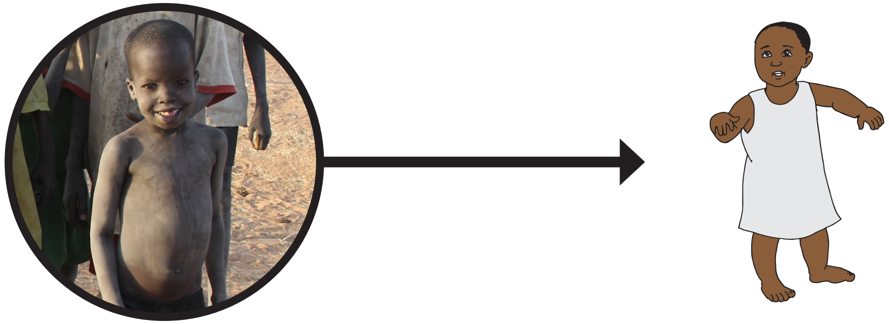

Suggested Training Activities

1. Show CHWs cards with the different age/sex icons and have them practice identifying them correctly.
2. Have CHWs practice identifying the image on the card and ticking the corresponding box on the individual register sheets.
3. Verbally give the CHWs different age/sex scenarios for the children they'll treat and have them mark the correct box on the register.

Materials Needed

1. Cards with the age/sex icons
2. Individual color copies of the register sheet
3. Pens

Patient Register: Medication: Amoxicillin

1. The pink box of amoxicillin is given to infants and the green box is given to toddlers. This corresponds to the correct dosage for infants and toddlers.

2. Mark the box below the image that matches the amoxicillin given to the child according to their age.

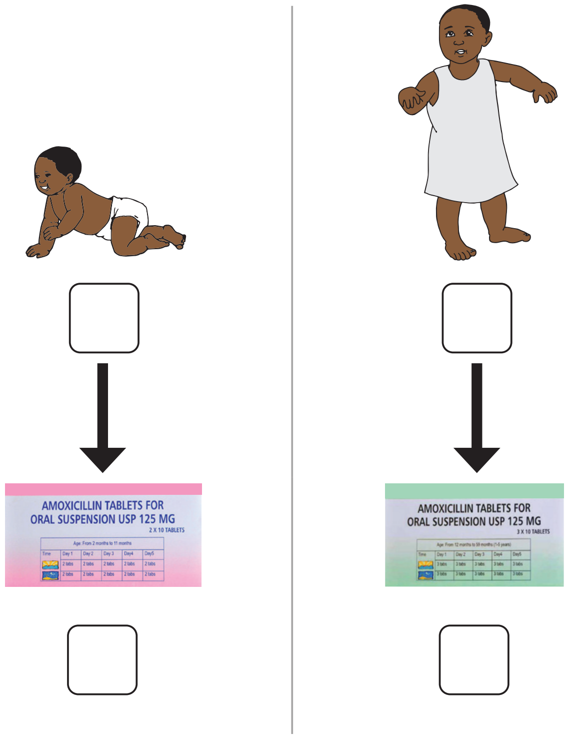

Suggested Training Activities

1. Give the CHWs different ages of children and ask them to mark which amoxicillin they would give the child.
2. Have the CHWs explain the difference between the pink and green boxes of amoxicillin.
3. Ask the CHW to explain how to give the amoxicillin.

Materials Needed

1. Boxes of amoxicillin for infants and toddlers
2. Individual color copies of the register sheet

Patient Register: Medication: Albendazole

1. Albendazole is only given to toddlers. (this is why the section is only below the toddler icon)

2. Half a pill is given to toddlers 1-2 years. A whole pill is given to toddlers 2 years or older.

3. If abendazole is given to the child, mark below the image that matches the albendazole given.

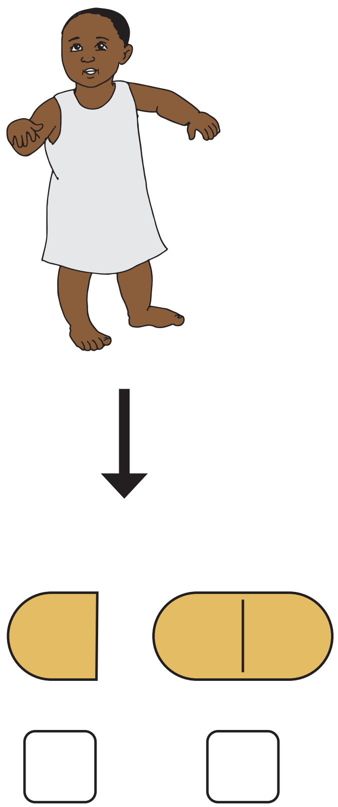

Suggested Training Activities

1. Give the CHWs different ages of children and ask them to mark which albendazole they would give to the child.
2. Show different cards with the medication images and ask CHWs to identify what drugs they are and who they are used for.
3. Ask the CHWs to come up with a rhyme or song to remember how to give the correct dosage of albendazole.

Materials Needed

1. Cards with the different medication icons
2. Individual color copies of the register sheet
3. Samples of albendazole

Patient Register: ID System

1. Carefully fold and tear the patient ID from the patient's page in the register and place it in a small plastic bag.

2. Give the ID card to the child's caregiver and explain to them to keep it in a safe place and bring it back each visit.

3. When the caregiver returns the next week, first open the register to the pages with the right color. Then match the colored bar of the same size and color on the register page.

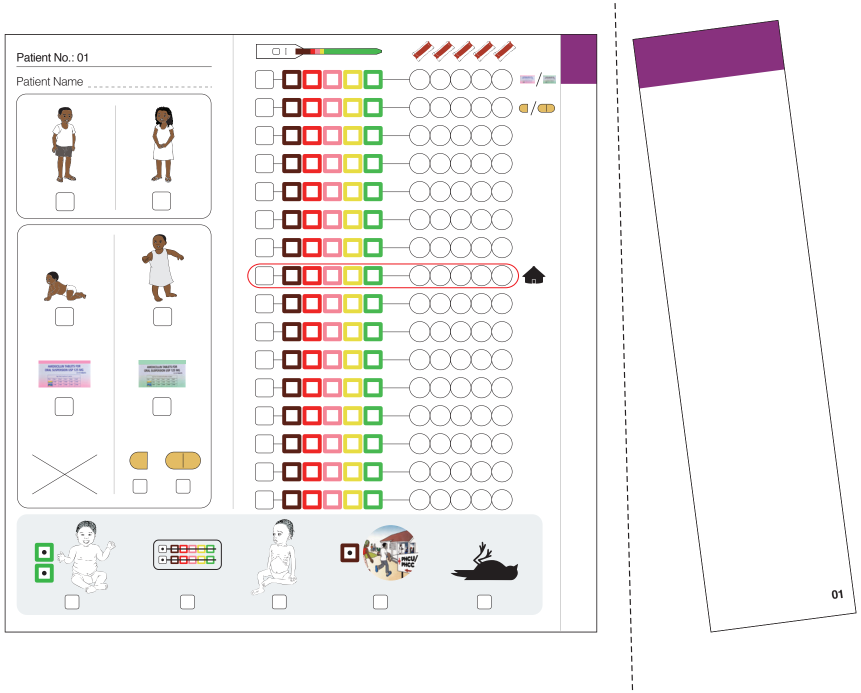

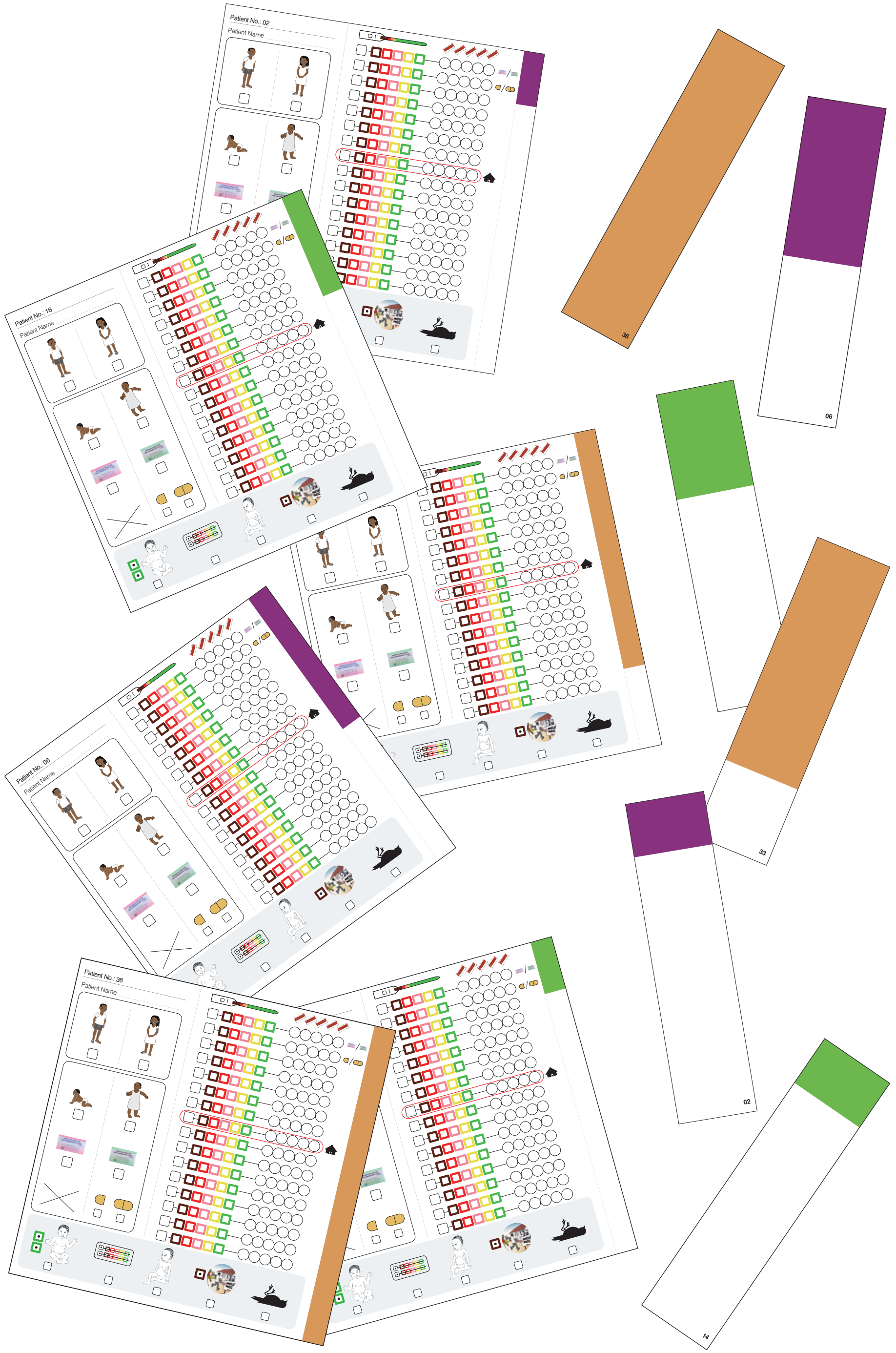

Suggested Training Activities

1. Separate a book of registers and patient IDs, give each CHW 3-5 patient IDs of different colors and ask them to find the corresponding register page.

Materials Needed

1. Full patient register

Patient Register: Status and treatment monitoring

1. Every week, mark the MUAC reading taken for the child and the RUTF dosage provided. One week = one row.

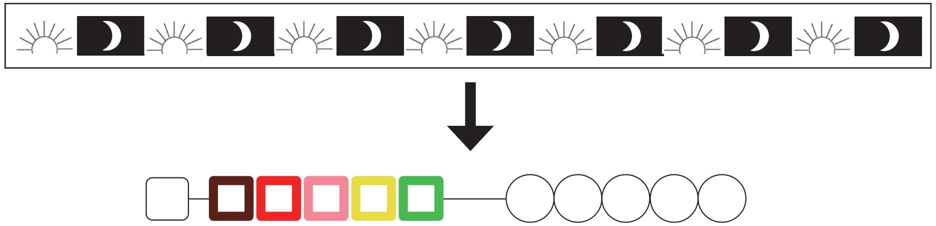

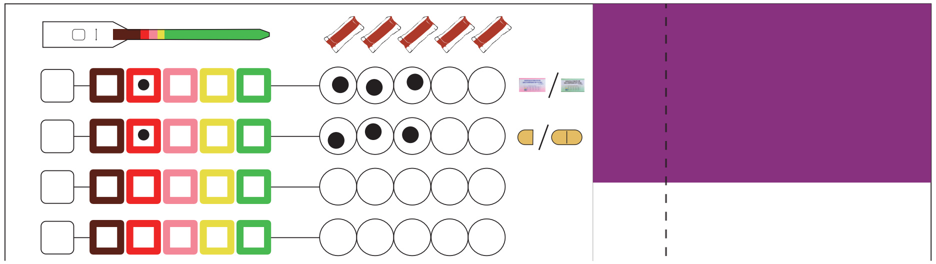

2. If the child is absent, mark the left most box and draw a line through the entire row. This reminds CHWs to skip to the next row when the child returns the following week.

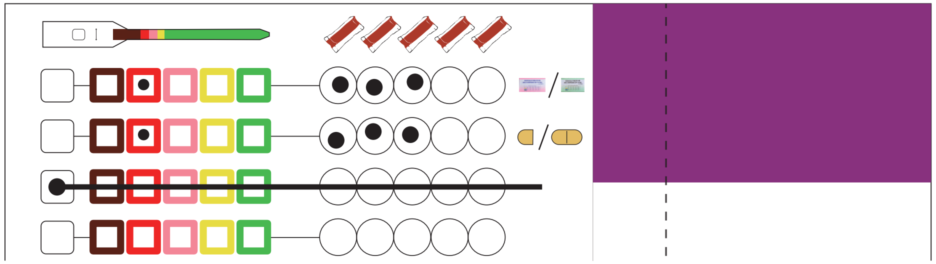

Suggested Training Activities

1. Practice counting out 16 weeks with the CHWs.
2. Prepare pieces of paper with the numbers 1-16 written on them. Have the CHWs take turns picking numbers out of a bag and pointing to the correct line on the register.
3. Draw a number to indicate the week and give the CHW a practice MUAC measurement and RUTF dosage to fill in.
4. Ask the CHWs to mark the next week as absent. Make sure they mark the correct row.

Materials Needed

1. Pieces of paper with the numbers 1-16 written on them
2. Individual color copies of the register sheet
3. Pens

Patient Register: Discharge Performance Indicators

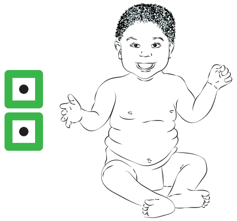

Cured
Child is in the green zone for the 2nd visit in a row and can be discharged.

This child is healthy and no longer malnourished.

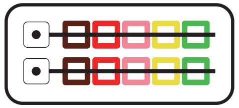

Default:
Child is absent for 2 weeks in a row and has dropped out of the program.

This child should be discharged. If the child returns, they can be readmitted.

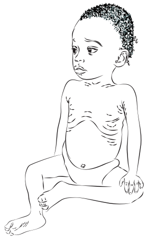

Non-responder:
Child is still malnourished after 16 weeks in the program.

This child should be discharged and referred.

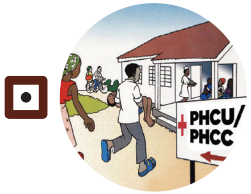

Transfer:
Child deteriorates into the dark red zone, develops a danger sign or doesn't have an appetite.

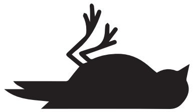

Death:
Child passes away while enrolled in the program.

Suggested Training Activities

1. Discuss the possible discharge outcomes that a CHW might encounter when a child is under their care.
2. Show the CHWs flash cards with the outcome icons and ask them to identify the outcome criteria (when will they know this child is cured, defaulted, non-respondent, transfer, etc.)
3. Ask them to identify the outcome and point to where they would make the mark on the register.

Materials Needed

1. Cards with outcome icons
2. Individual color copies of the register sheet

1.4
Key RUTF Feeding Messages

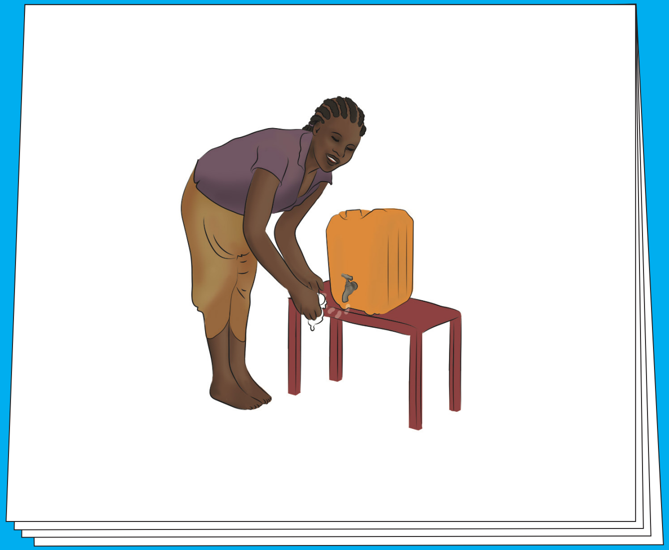

Flipchart:
Key RUTF Feeding Messages

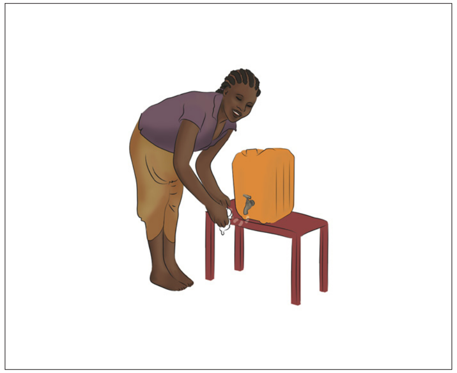

1. Wash your hands with soap and water before giving RUTF.

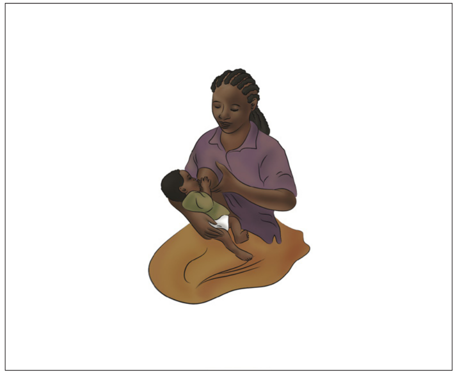

2. Before giving RUTF, breastfeed your child.

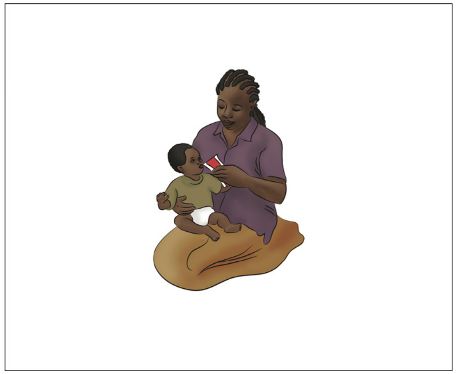

3. After breastfeeding, feed your child as much RUTF as she/he can eat

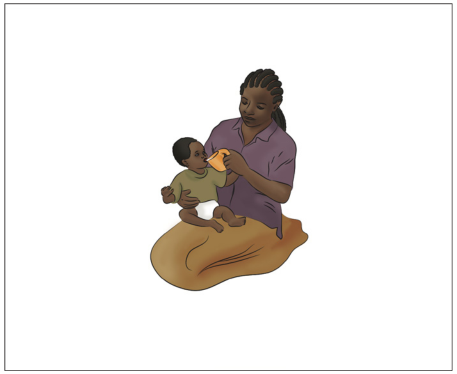

4. Give your child plenty of clean water to drink.

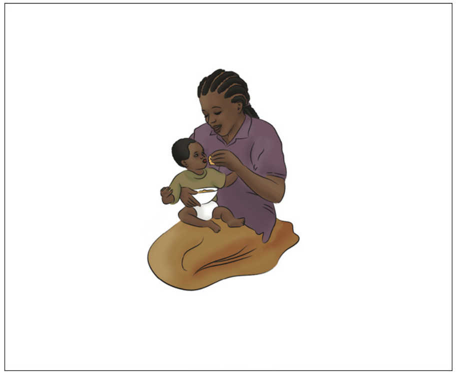

5. If your child is still hungry, feed your child family food. Make sure your children eat the correct amount of RUTF before giving additional food.

Suggested Training Activities

1. Ask the CHWs to describe what is happening in the images, then explain the message when the discussion is finished.

Materials Needed

1. Flipcharts

Part 2: Essential Procedures

2.1 Assessment of Oedema

Danger Signs: Assessment of Oedema

1. Press thumbs on the top of both feet for 3 seconds (use locally appropriate words or song to help count to 3 seconds.

2. Only light pressure is required, as if pressing into a ripe mango.

3. If there is an indent like seen in the photo below in both feet after you lift your thumbs, then the child has oedema and needs to be referred immediately.

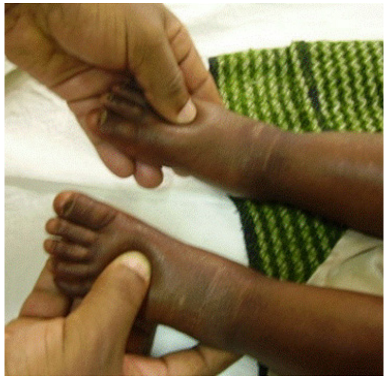

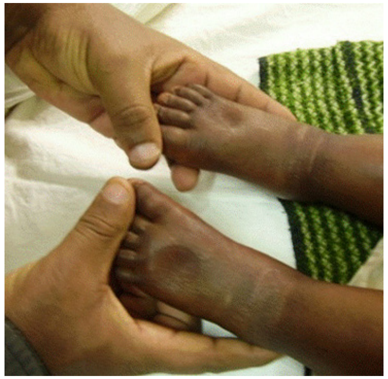

Suggested Training Activities

1. Have the CHWs practice on each other's feet. Trainers should check to make sure they are applying the correct amount of pressure for 3 seconds

2.2 Appetite Test

Danger Signs: Appetite test

1. The caregiver should wash his/her hands.

2. Find a quiet area to conduct the test.

3. Have the caregiver offer the RUTF from the packet or if the child is too small or weak, caregiver can use his/her hands to feed the child.

4. The caregiver should encourage the child to eat and offer the RUTF gently. Do not force the child to eat the RUTF. Be patient and observe, the test may take up to 30 minutes.

5. Have plenty of water for the child to drink while he/she is taking RUTF.

If the child eats more than 1/3 of the sachet, they pass

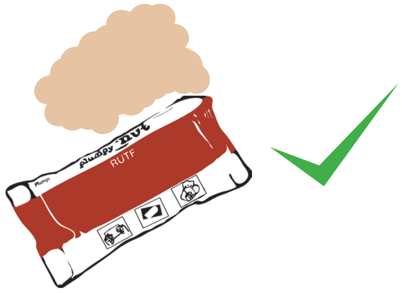

If the child eats less than 1/3 of the sachet, they fail and need to be referred

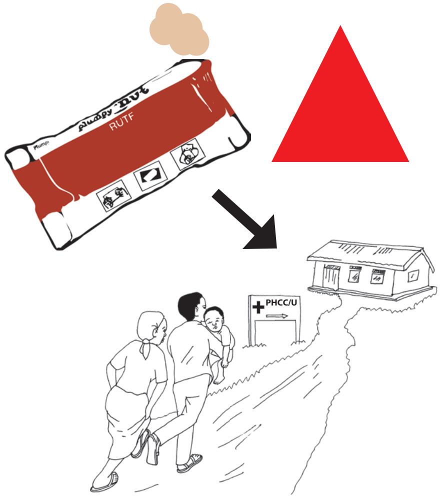

Suggested Training Activities

1. Show the CHWs a picture of a goat (or other commonly eaten food). If they were to eat the goat over three days, how would they divide the goat so they would have an equal amount each day? Have the CHWs draw with a pen how they would divide the goat into 3. Now how would they divide the RUTF sachet into three equal portions?
2. Give each CHW an image of a sachet with RUTF and ask them to divide it into three equal parts with a pen.
3. Have CHWs observe during an OTP day how to conduct an appetite test. Pass around the sachet once the child has completed the test so the CHWs know how it should look and feel.

Materials Needed

1. Image of sachet of RUTF
2. Images of goats or other commonly eaten items
3. Pens

2.3
Danger Signs

Refer:

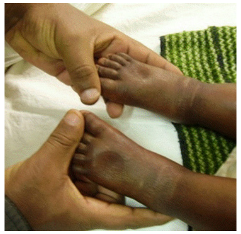

Bilateral pitting oedema present

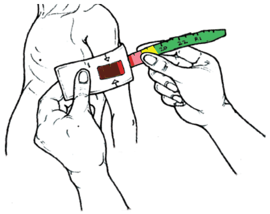

Dark red MUAC

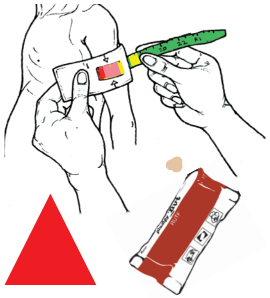

Red/Pink MUAC plus danger signs or poor appetite

Treat:

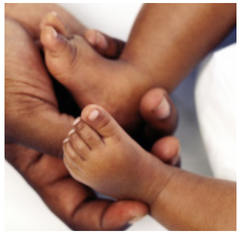

No bilateral pitting oedema present

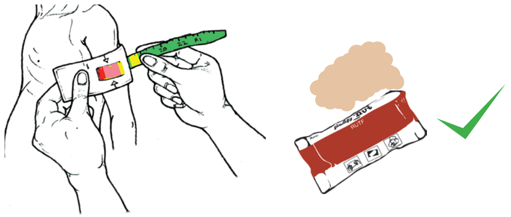

Red/Pink MUAC and no danger signs and good appetite

Danger Signs:
Step 1: Look

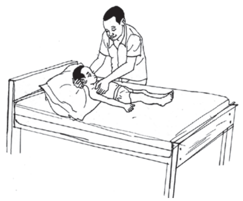
Stiff neck

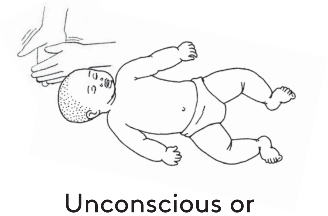
Unconscious or
abnormally sleepy

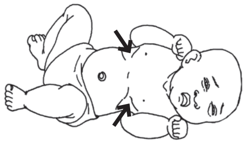
Chest in-drawing

Danger!

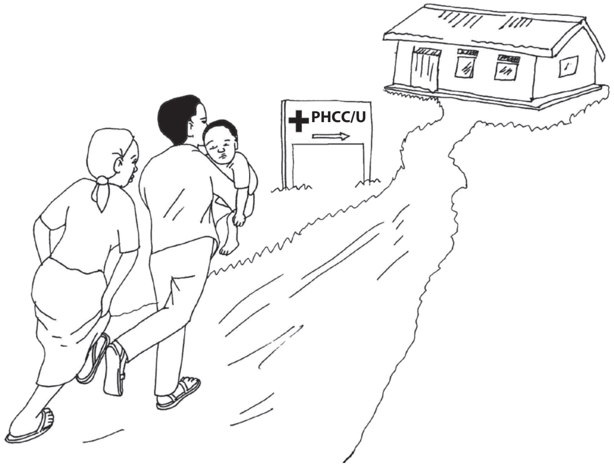

Refer child to health facility
immediately

Danger Signs:
Step 2: Ask

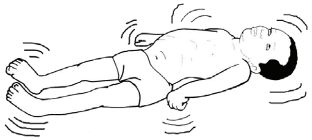
Convulsions

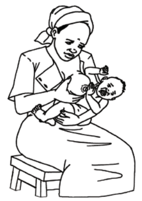
Unable to
breastfeed or drink

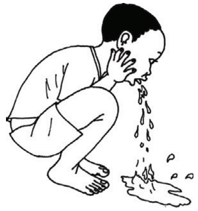
Vomits everything

Danger!

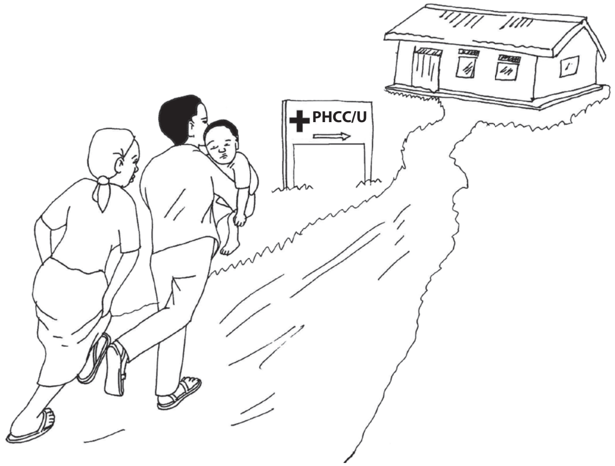

Refer child to health facility
immediately

Danger Signs: Step 3: Assess

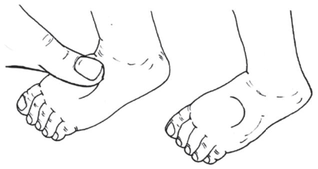

Swelling of both feet

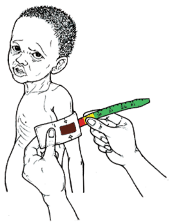

Dark Red MUAC

Danger!

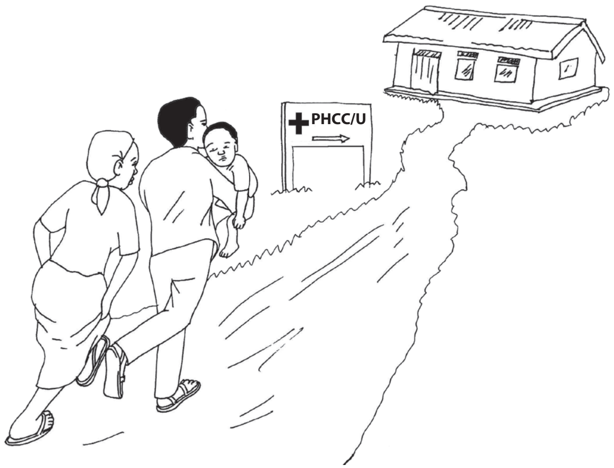

Refer child to health facility immediately

Suggested Training Activities

1. Ask the CHWs to list the danger signs.
2. Ask the CHWs to describe how they can identify the danger signs.

Materials Needed

1. iCCM job aid with danger signs

Part 3: Simplified SAM Treatment Protocol

Simplified SAM Treatment Algorithm:

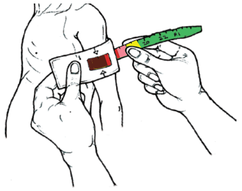

or
other danger signs

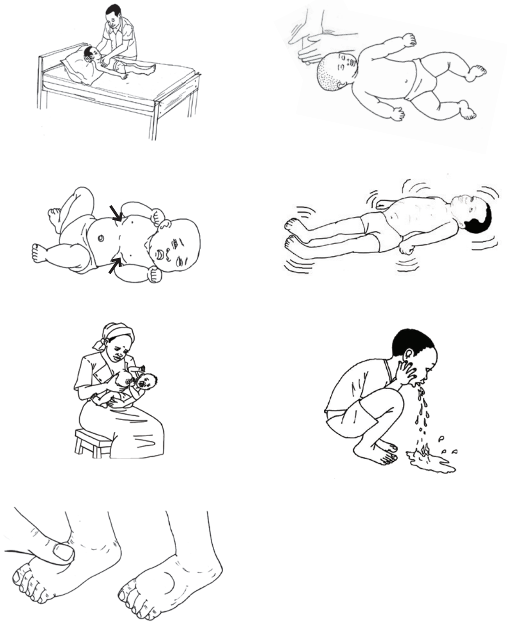

Referral to health facility

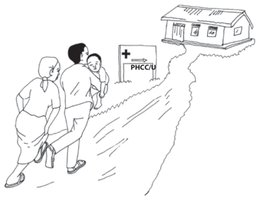

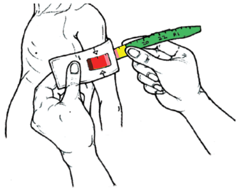

Appetite Test

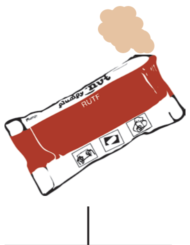

Pass

Fail

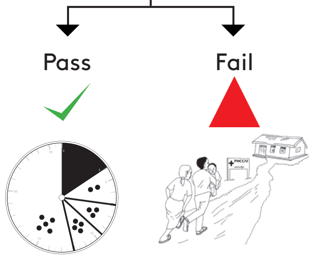

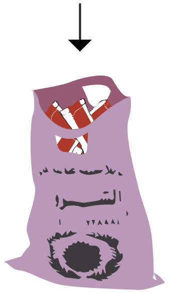

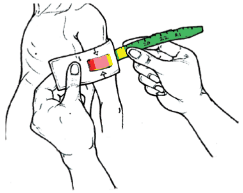

Appetite Test

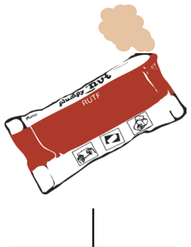

Pass

Fail

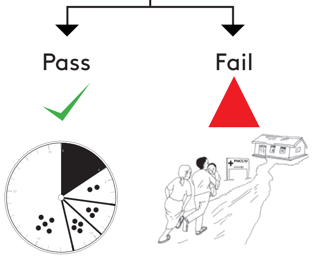

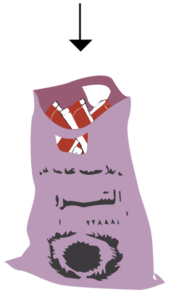

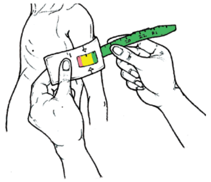

Referral to TSFP

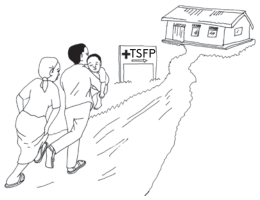

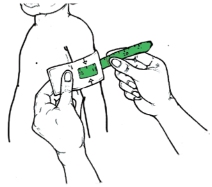

Normal

Tell the caregiver their child is not malnourished and encourage them to continue feeding their child the same way.

3.1 First Visit (Week 1)

First Visit: Assess

1. Welcome the caregiver and child

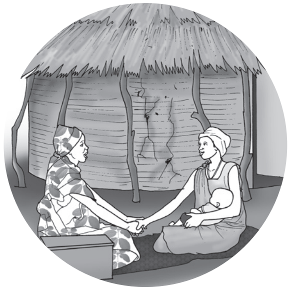

2. Ask for the child's name and age. (confirm child is between 6-59 months)

3. Ask for the reason the child is here today.

4. Assess for general danger signs, refer immediately if they exist.

3. General Danger Signs

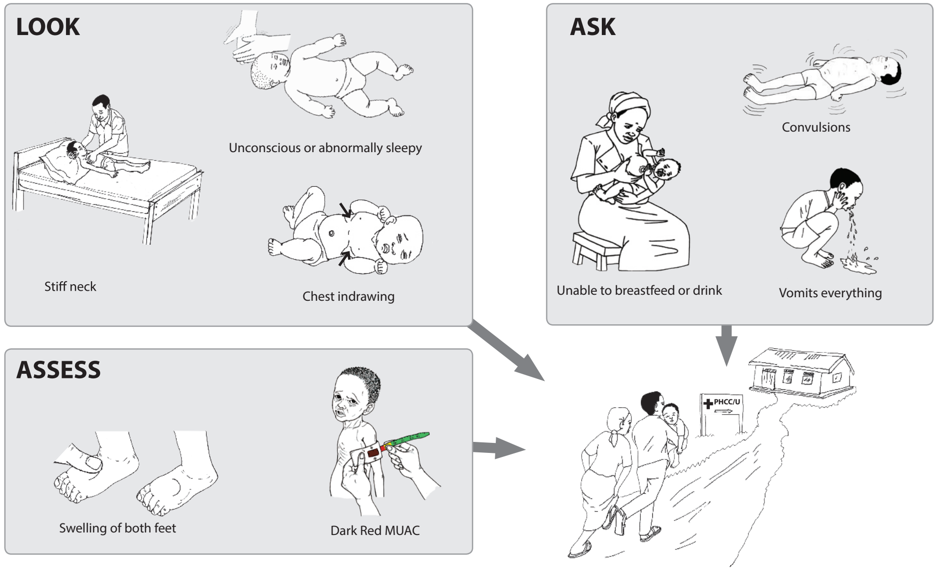

First Visit: Assess

5. If no danger signs exist, take the MUAC of the child.

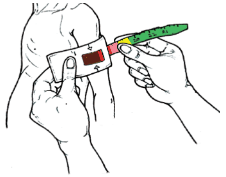

If MUAC is dark red, refer immediately

Referral to health facility

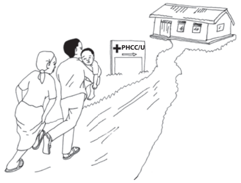

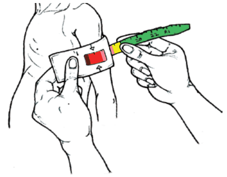

If MUAC is red, test appetite first

Appetite Test

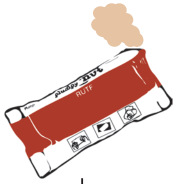

Pass

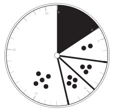

Weigh Child

Fail

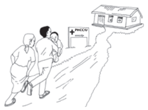

Referral to health facility

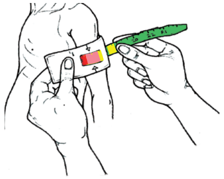

If MUAC is pink, test appetite first

Appetite Test

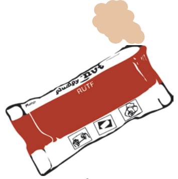

Pass

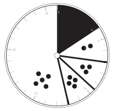

Weigh Child

Fail

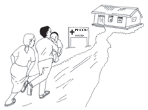

Referral to health facility

If the child has a red or pink MUAC and a poor appetite, refer immediately.

If the child has a red or pink MUAC and a good appetite, you will weigh and treat. Open the register to the next blank page and start treatment. (see next page)

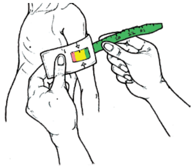

If MUAC is yellow, refer to TSFP

Referral to TSFP

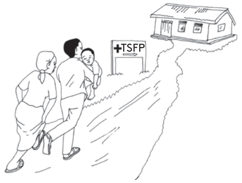

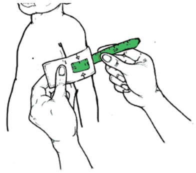

Normal

Tell the caregiver their child is not malnourished and encourage them to continue feeding their child the same way.

Suggested Training Activities

1. Use the training cards to draw different combinations of danger signs, MUAC measurements, and appetite test results. Role play with the CHWs to see if they would treat, refer, or need more information to decide
2. Color/Outcome association: draw a card from a deck of cards with MUAC colors. Ask CHWs what the associated action step would be.
3. Ask CHWs to arrange the 5 MUAC colors in order of severity.

Materials Needed

1. Cards with the 5 MUAC colors, danger signs, appetite test results

First Visit: Admit and Treat

1. If the child has no danger signs, a red or pink MUAC, passes the appetite test, and is not in the black zone then admit the child for treatment.

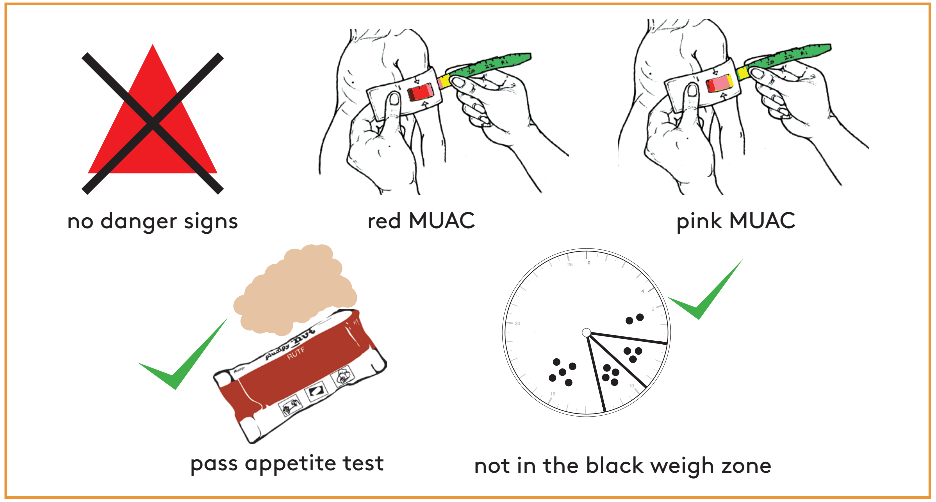

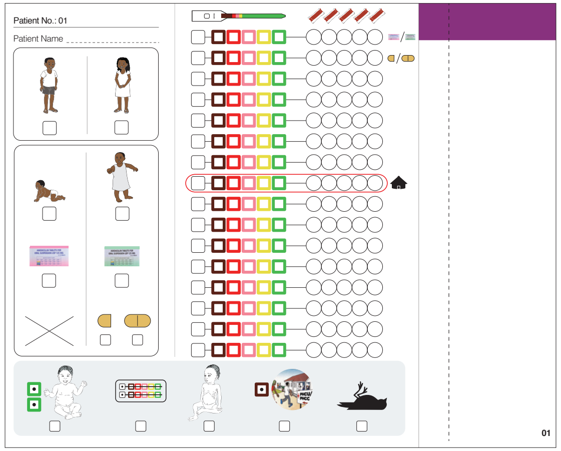

admit for treatment

First Visit: Treat

2. Record the child's information on the register: sex, age, MUAC color, and dosage scale reading.

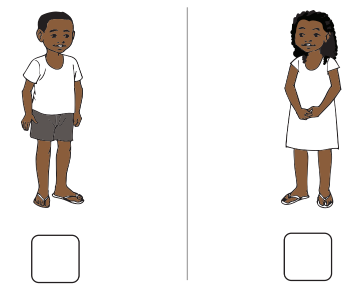

Record child's sex

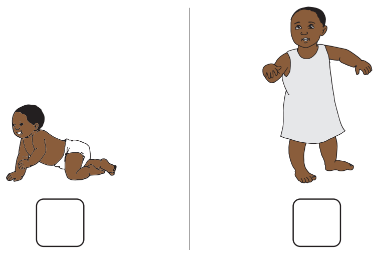

Record child's age

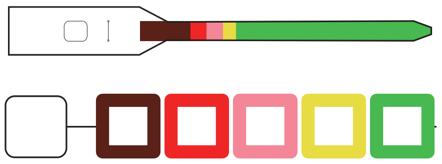

Record MUAC color

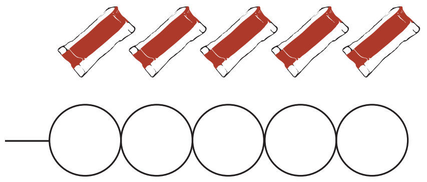

Record dosage scale reading

3. Count out the week's dose of RUTF using the dosage calculator. Remove one sachet for the appetite test.

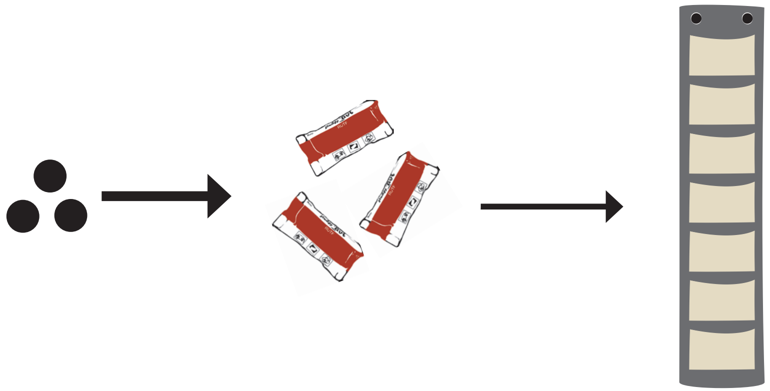

4. Administer amoxicillin based on the child's age and record.

Advise caregiver how to correctly give amoxicillin at home. It is very important the child takes the entire dose until finished.

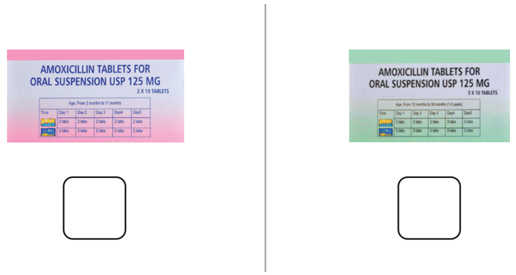

Amoxicillin is given to every severely malnourished child that you treat on the day of admission regardless of whether a child has pneumonia or not.

First Visit: Treat

5. Counsel the caregiver on how to give RUTF at home using the flipchart. Ask the mother if she has any questions.

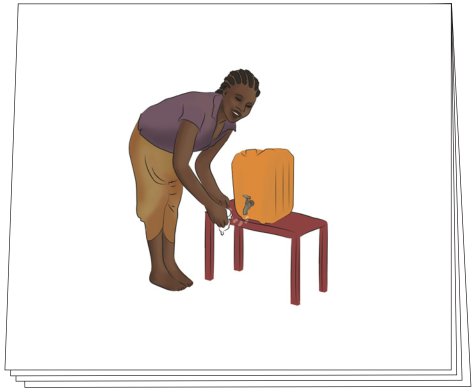

6. Give the RUTF dosage and ID card to the caregiver and instruct her to return again in 7 days.

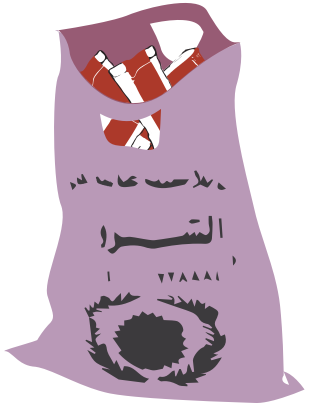

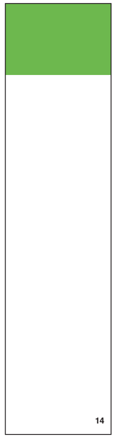

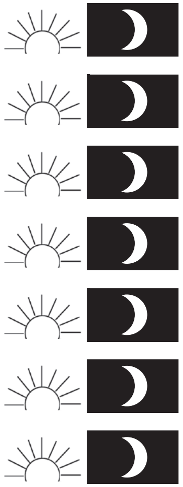

3.2 Week 2

Week 2: Assess

1. Match the ID with the correct page in the register.

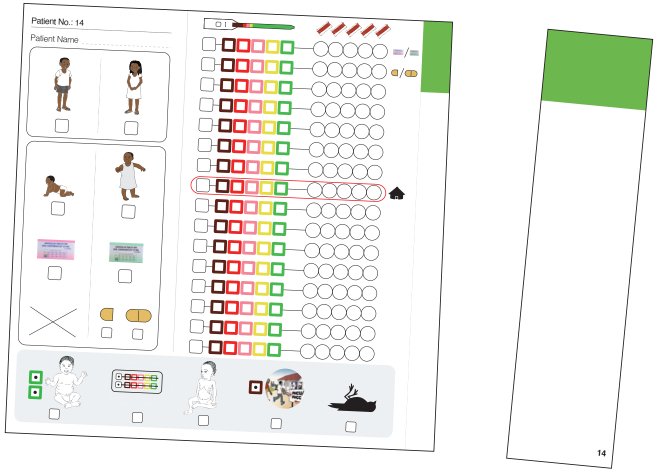

2. Ask how the child was in the past week and whether they finished all the RUTF.

3. Assess for general danger signs.

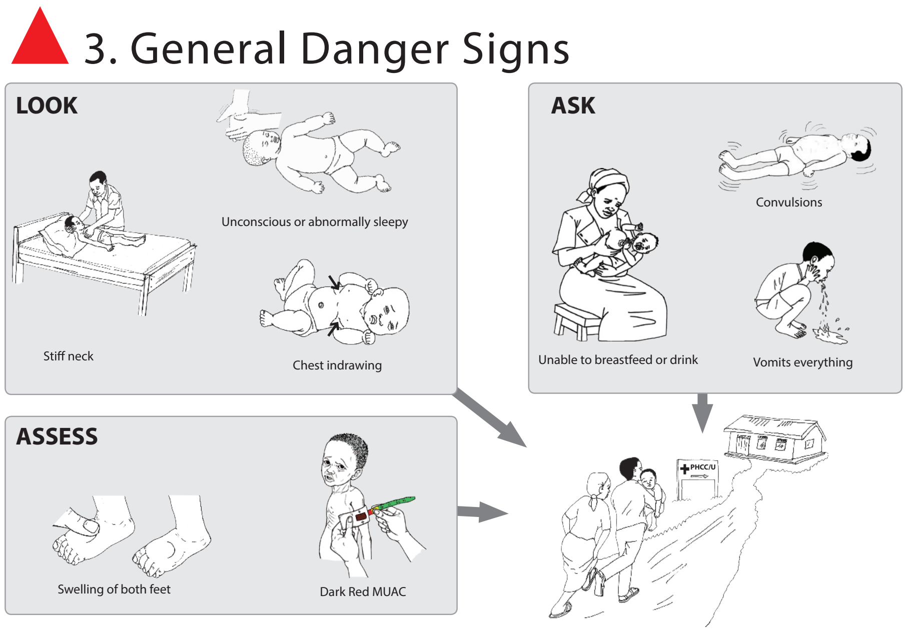

4. Conduct the appetite test with one sachet of RUTF.

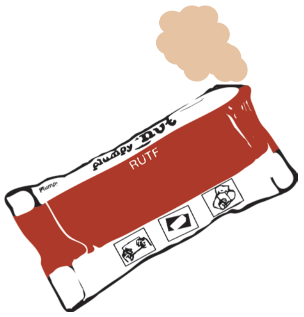

Week 2: Treat

1. If the child has no danger signs and passes the appetite test, weigh the child and continue treatment.

2. Record the second week's measurements on the register: MUAC color and dosage scale reading.

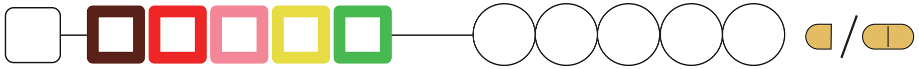

3. Count out the week's dose of RUTF using the dosage calculator. Remove one sachet for the appetite test.

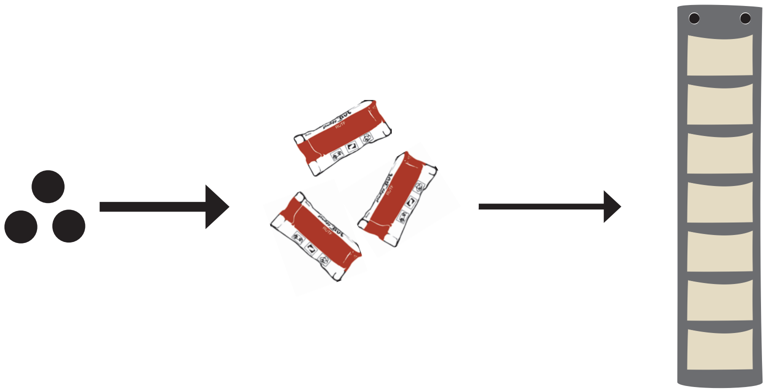

4. Administer albendazole based on the child's age and record.

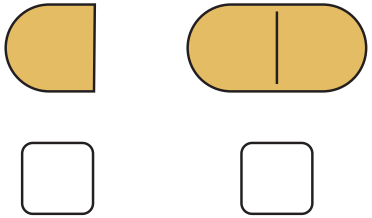

5. Counsel the caregiver on how to give RUTF at home using the flipchart. Ask the mother if she has any questions.

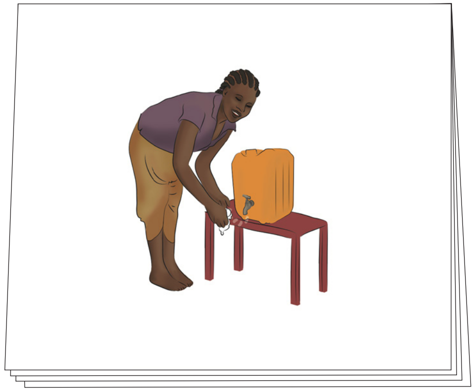

6. Give the RUTF dosage and ID card to the caregiver and instruct her to return again in 7 days.

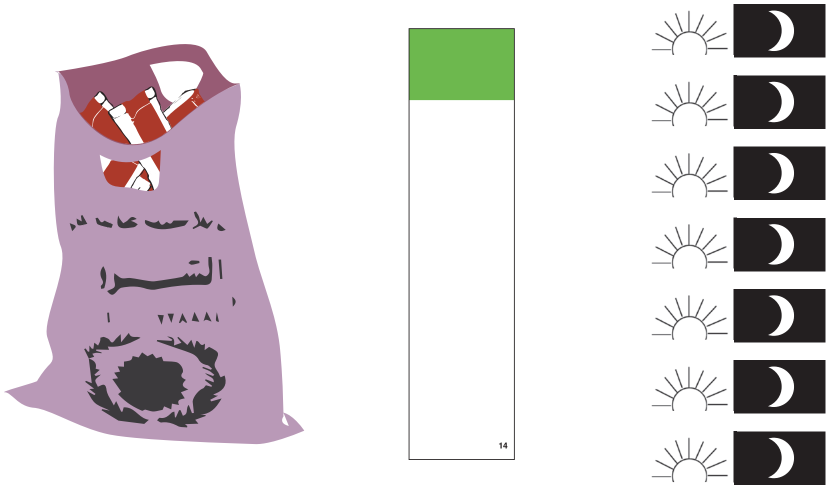

3.3
Week 3 - 16

Week 3 - 16: Assess

1. Match the ID with the correct page in the register.

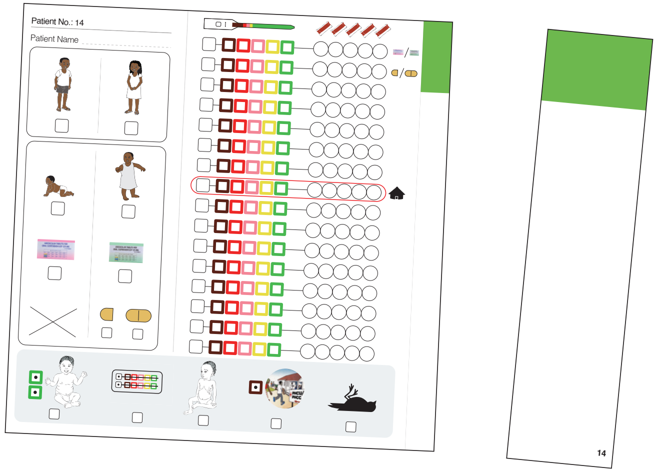

2. Ask how the child was in the past week and whether they finished all the RUTF.

3. Assess for general danger signs.

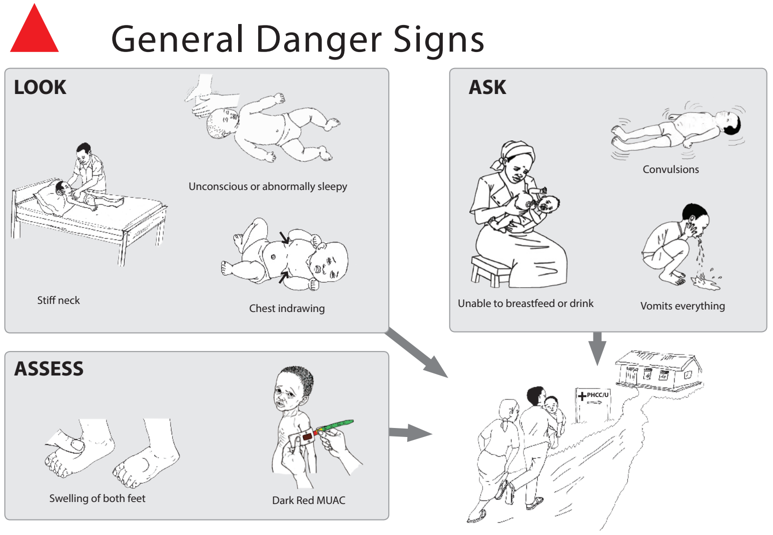

4. Conduct the appetite test with one sachet of RUTF.

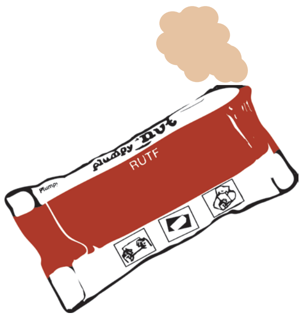

Week 3 - 16: Treat

1. If the child has no danger signs and passes the appetite test, weigh the child and continue treatment.

2. Record the third week's measurements on the register: MUAC color and dosage scale reading.

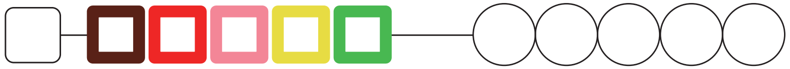

3. Count out the week's dose of RUTF using the dosage calculator. Remove one sachet for the appetite test.

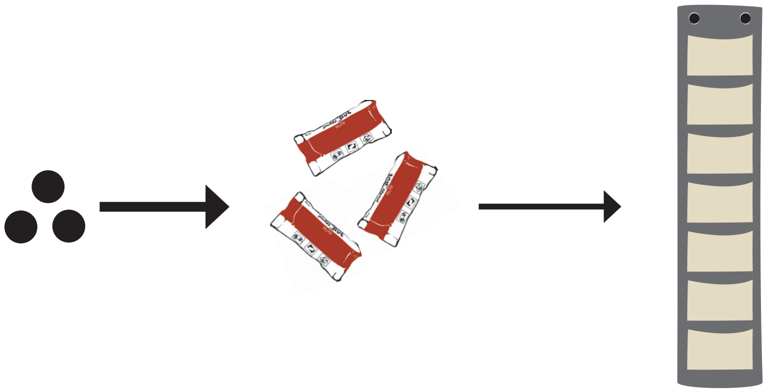

4. Give no medication, but in week 4 refer the child to the health facility for measles vaccination.

5. Counsel the caregiver on how to give RUTF at home using the flipchart. Ask the mother if she has any questions.

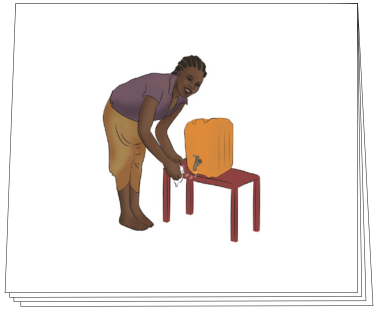

6. Review the child's record to see if any outcomes or home visits apply.

If no: give the RUTF dosage and ID card to the caregiver and instruct her to return again in 7 days.

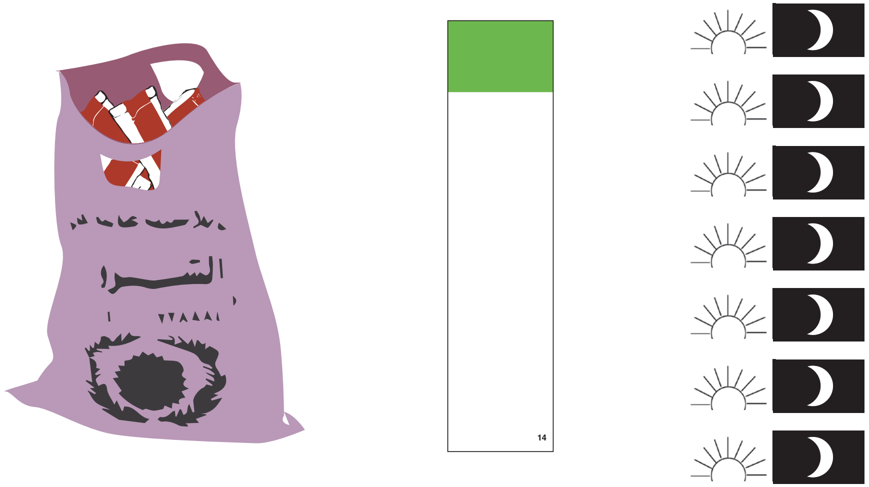

If yes:

1. Explain the outcome to the caregiver
2. Give any final rations or referral cards
3. Record the outcome

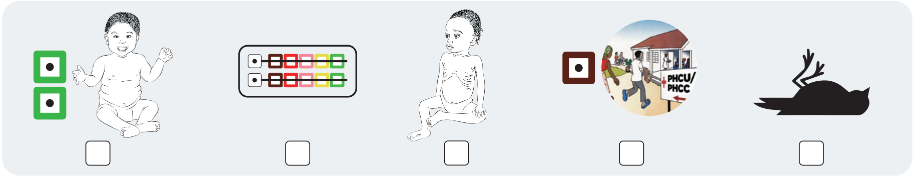

3.4
Discharge Criteria

Discharge Criteria:

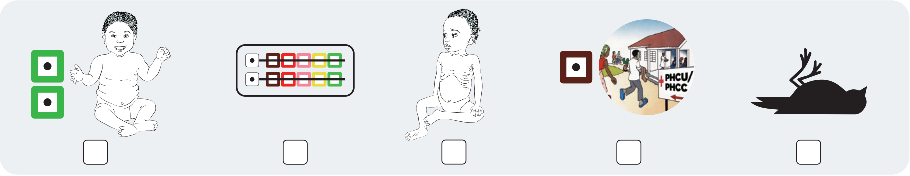

1. This information can only be entered when one of the 5 outcomes has been reached.

2. Once a child has readings which match one of the status options, mark the appropriate box below the image that matches the child's status. This child is then discharged from the program.

3. Once an outcome has been selected, the patient register is closed and no further changes should be made.

4. If the child becomes severely malnourished again in the future, a new page in the register will need to be made and the child will need to be readmitted.

Suggested Training Activities

1. Prepare flash cards with outcome icons to practice identification.
2. Practice recording different scenarios, going through all the outcomes.
3. Have CHWs take a filled out register and present the case.
4. Give CHWs an outcome and have them fill out to register - How does the register need to look in order to reach that outcome?
5. Have CHWs explain, then act out, what they have to do on a weekly basis when treating for malnutrition (asses for danger signs, measure MUAC, give appetite test) vs. what they have to do only in week 1 (start a new register page, give amoxicillin) and week 2 (give albendazole to toddlers)

Materials Needed

1. Cards with outcome icons
2. Individual copies of the register sheets (blank)
3. Individual copies of the register sheets (filled in with different scenarios)

Special Cases

1. If: Child has a dark red MUAC and refuses to go to the health facility for treatment, explain to the caregiver that the child may not recover with RUTF since they have medical complications the CHW cannot treat and likely no appetite. If they still refuse, take action.

Action: Treat by CHW following the protocol for red/pink MUAC admission

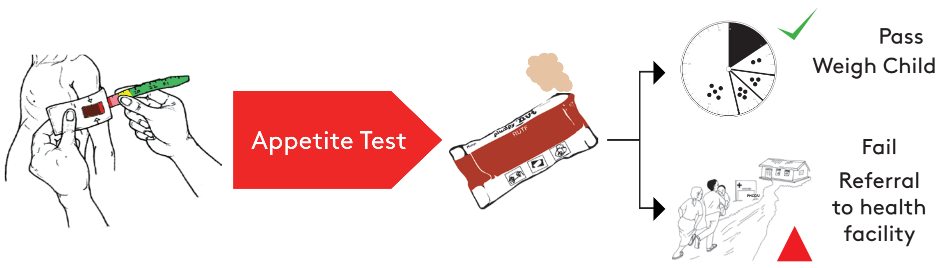

2. If: Child is under treatment and develops a danger sign at any time.

Action: Refer to health facility

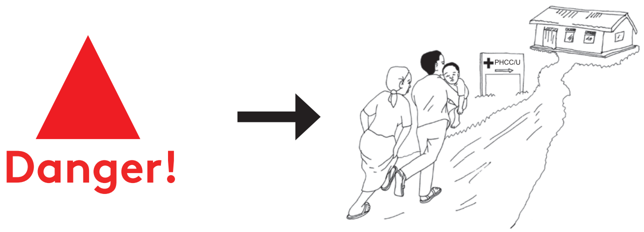

3. If: Child is under treatment and falls in the dark red zone.

Action: Refer to health facility

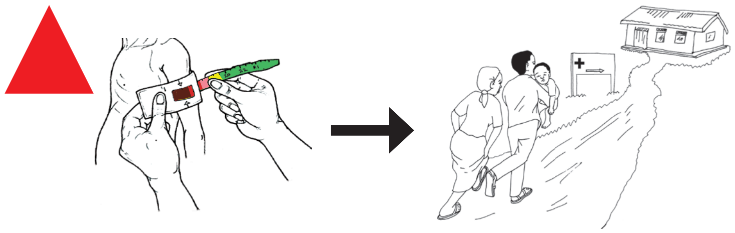

4. If: Child is under treatment and misses a visit.

Action: Make a home visit and encourage the caregiver to return

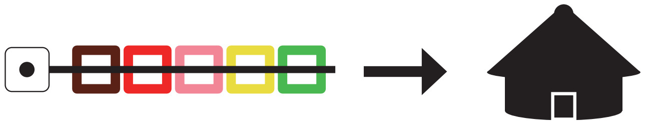

5. If: Child is under treatment and has 3 successive visits in the same zone.

Action: Home visit. Ask the caregiver to show the remaining sachets of RUTF and watch the child eat a sachet. Advise the caregiver not to share the RUTF with others.

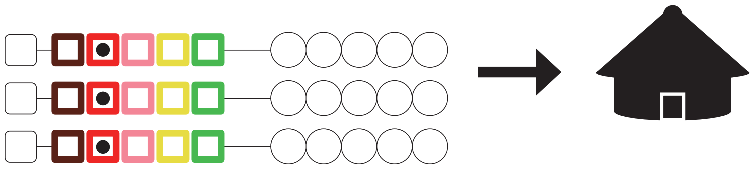

6. If: Child is under treatment and has 4 successive visits in the same zone.

Action: Referral to health facility

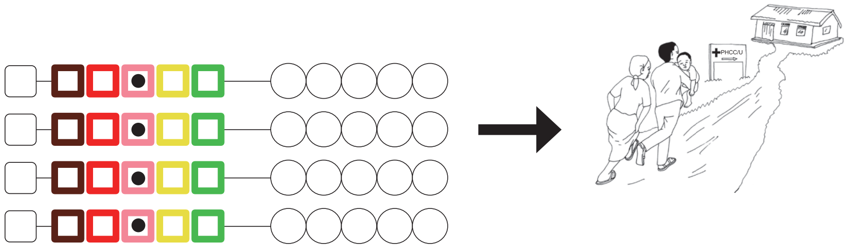

Home Visits:

Tasks:

1. Remind the caregiver to give the child plenty of clean water to drink and to wash hands before giving the RUTF.

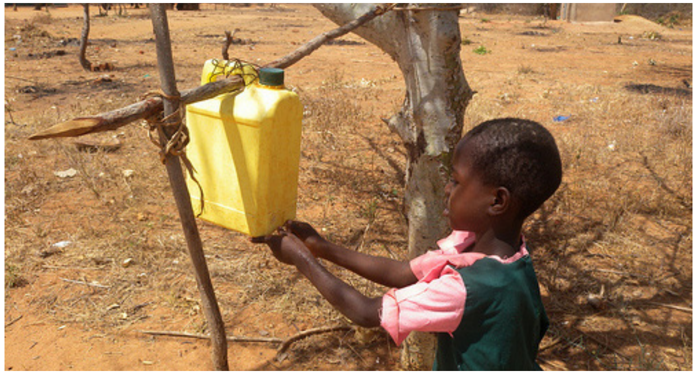

2. Ask the caregiver to show the remaining sachets of RUTF and watch the child eat a sachet. See if the correct number of sachets remains. Advise the caregiver not to share the RUTF with others.

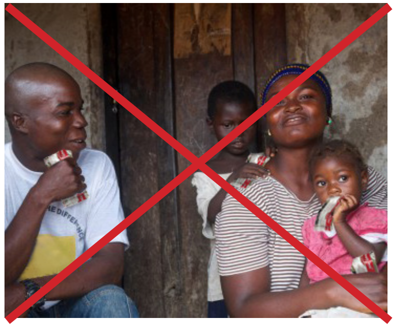

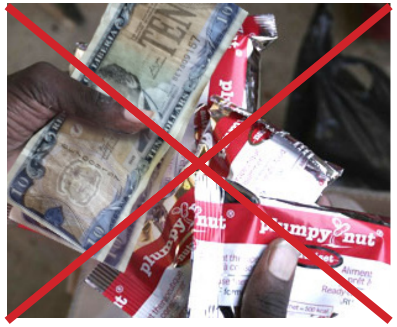

3. Determine the reasons for missing weekly visits.

4. Counsel families on the importance of the treatment regimen.

Part 4:
iCCM + SAM
Treatment Protocol

Malaria + SAM

1. Fever or history or fever in the last 3 days.

2. SAM.

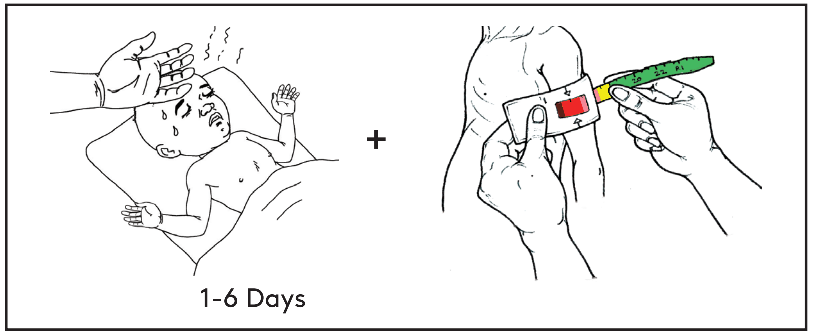

1-6 Days

Treat with ACT according to iCCM protocol and RUTF according to dosage scale

AGE	TREATMENT	DAY 1	DAY 2	DAY 3	DAY 4	DAY 5	DAY 6	DAY 7
6-12 months								
1 - 5 years								

1. Fever for more than 7 days

2. SAM.

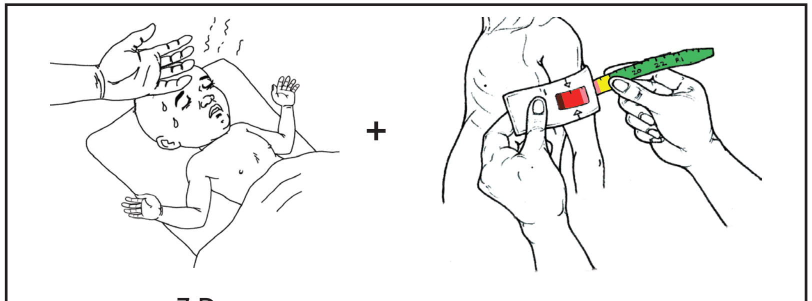

7 Days

Refer with Pre-referral Treatment

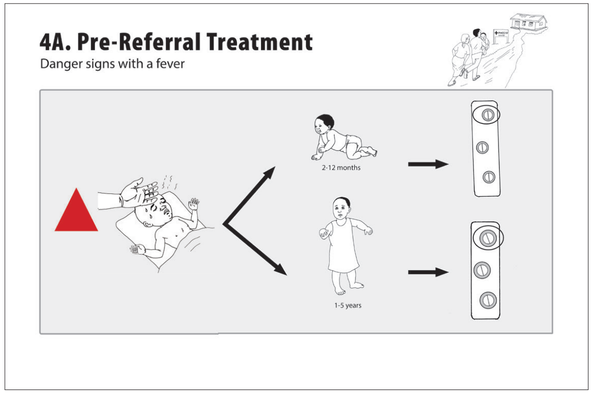

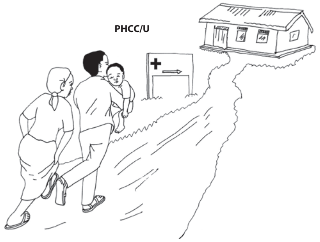

Pneumonia + SAM

1. Cough with fast breathing for fewer than 21 days.

2. SAM.

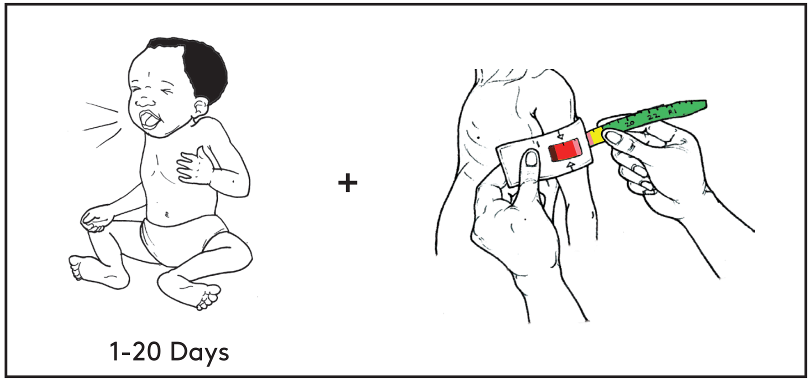

ONE dose of amoxicillin at admission depending on the child's age + RUTF according to dosage scale.

AGE	TREATMENT	DAY 1		DAY 2		DAY 3		DAY 4		DAY 5		DAY 6		DAY 7	
6 -12 months															
1 – 5 years															

1. Cough with fast breathing for more than 21 days.

2. SAM.

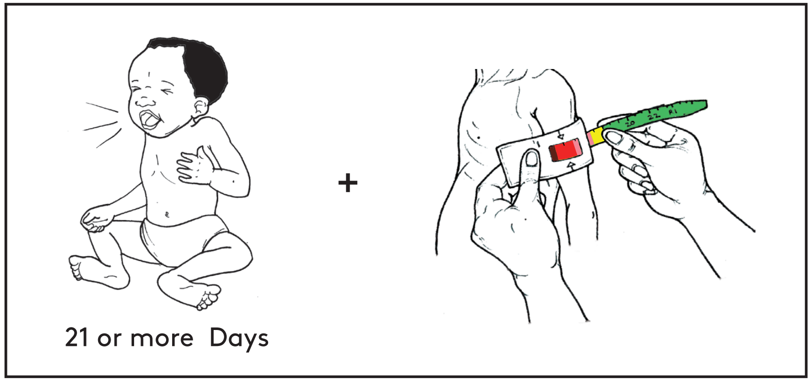

Refer with Pre-referral Treatment

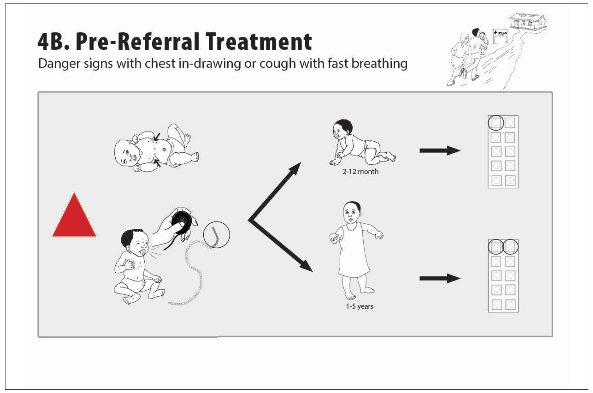

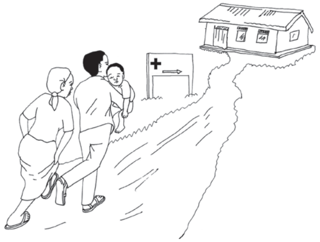

Diarrhea + SAM

1. Diarrhea for fewer than 14 days.

2. SAM.

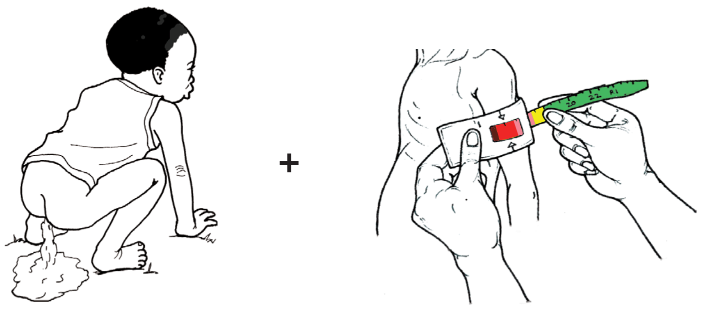

1-13 Days

Do not give ORS or ZINC tablets, these nutrients can be found in RUTF. Advise the caregiver to breastfeed and give RUTF.

AGE	TREATMENT	DAY 1	DAY 2	DAY 3	DAY 4	DAY 5	DAY 6	DAY 7
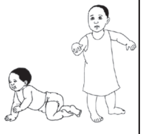 7 months -5 years	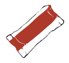	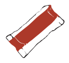	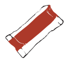	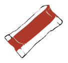	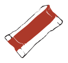	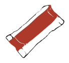	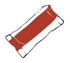	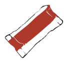

1. Diarrhea for more than 14 days.

2. SAM.

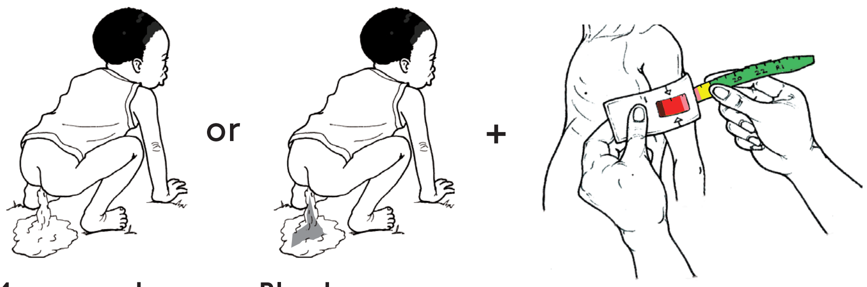

14 or more days **Blood or worms**

Refer with Pre-referral Treatment

Do not give ORS. Instead, give the child sugar water.

(4 heaping teaspoons of sugar dissolved in a cup of clean water)

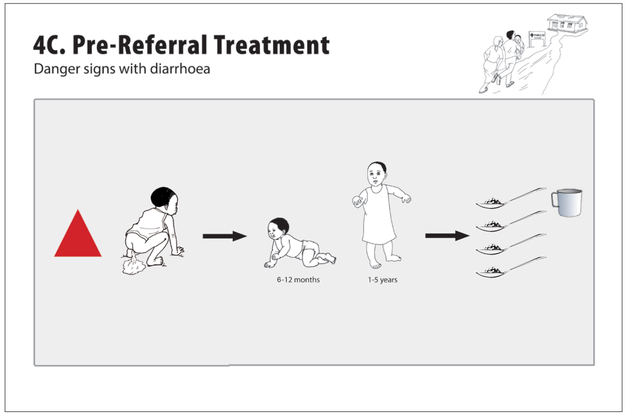

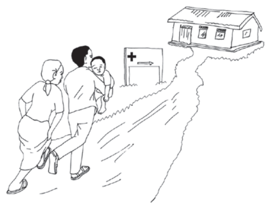

Suggested Training Activities

1. Prepare different scenarios with combinations of pneumonia, diarrhea, malaria, and SAM, with and without danger signs. Ask the CHWs to describe the steps for treatment or referral.
2. Have the CHWs role play with each other, practicing the assessment steps and administering medication.

Materials Needed

1. iCCM/nutrition assessment and treatment materials

INTERNATIONAL RESCUE COMMITTEE
